# Benefits and Detriments of Gadolinium from Medical Advances to Health and Ecological Risks

**DOI:** 10.3390/molecules25235762

**Published:** 2020-12-07

**Authors:** Colin Unruh, Nicolas Van Bavel, Max Anikovskiy, Elmar J. Prenner

**Affiliations:** 1 Department of Biological Sciences, University of Calgary, Calgary, AB T2N 1N4, Canada; colin.unruh@ucalgary.ca (C.U.); nicolas.vanbavel@ucalgary.ca (N.V.B.); 2 Department of Chemistry, University of Calgary, Calgary, AB T2N 1N4, Canada

**Keywords:** gadolinium, chelates, MRI, membranes, membrane-metal interactions, environmental impact, remediation, gadolinium exposure, gadolinium deposition, surface potential

## Abstract

Gadolinium (Gd)-containing chelates have been established as diagnostics tools. However, extensive use in magnetic resonance imaging has led to increased Gd levels in industrialized parts of the world, adding to natural occurrence and causing environmental and health concerns. A vast amount of data shows that metal may accumulate in the human body and its deposition has been detected in organs such as brain and liver. Moreover, the disease nephrogenic systemic fibrosis has been linked to increased Gd^3+^ levels. Investigation of Gd^3+^ effects at the cellular and molecular levels mostly revolves around calcium-dependent proteins, since Gd^3+^ competes with calcium due to their similar size; other reports focus on interaction of Gd^3+^ with nucleic acids and carbohydrates. However, little is known about Gd^3+^ effects on membranes; yet some results suggest that Gd^3+^ interacts strongly with biologically-relevant lipids (e.g., brain membrane constituents) and causes serious structural changes including enhanced membrane rigidity and propensity for lipid fusion and aggregation at much lower concentrations than other ions, both toxic and essential. This review surveys the impact of the anthropogenic use of Gd emphasizing health risks and discussing debilitating effects of Gd^3+^ on cell membrane organization that may lead to deleterious health consequences.

## 1. Introduction to Gadolinium

Gadolinium (Gd), an element with the atomic number 64, belongs to a group of atoms called lanthanides or rare earth metals. The former term is certainly a misnomer considering that these elements are present in fairly plentiful amounts in nature and some of them (e.g., cerium) are even more abundant compared to common cobalt, lead, zinc or gold [1]. The series contains 15 elements starting with lanthanum having the atomic number 57 through lutetium with the atomic number 71. In nature, lanthanides do not exist in pure form but rather are found in phosphate or carbonate minerals including samarskite, monazite, and bastnaesite [2]. Mining of these ores is mainly done in China, although reserves also exist in large amounts in Brazil, Vietnam, Russia, and India, with smaller reserves in the United States, Australia, Greenland, and Tanzania [3].

Yttrium was the first discovered lanthanide. Gd, the focus of this review, was described almost a century later, in 1880, by the Swiss chemist Marignac, who observed new spectroscopic lines in a mineral. For more information on the discovery of lanthanides, an interested reader is referred to a comprehensive book by Evans [4]. Chemistry came a long way and the widespread use of analytical techniques in a modern-day laboratory overshadows the meticulous work required in the earlier times to extract and isolate these elements. One statement from the book clearly illustrates this point—“some of the rarer lanthanides required as many as 40,000 fractional crystallizations before they were really pure”, and crystallization was the only purification method utilized until the first half of the 20th century.

Furthermore, purification of these elements was naturally obstructed due to their structural similarity. The atoms only differ in the number of electrons occupying the 4f orbitals, which are not outer shells [5]. Such a unique electronic configuration exerts a twofold effect on the behavior of lanthanides. On one hand, the electrons in the 4f orbitals make the elements spectroscopically active, endowing them with strong magnetic susceptibility, i.e., the ability to magnetize in an external magnetic field, and capacity to absorb and emit light. On the other hand, the 4f electrons remain shielded by the outer shell electrons even upon ionization or complexation with ligands, keeping the spectroscopic properties of lanthanides highly conserved.

In a nutshell, Gd magnetic susceptibility arises due to seven unpaired electrons in the 4f orbitals. This configuration is shared by only one other element in the series, europium. As a trivalent ion, Gd^3+^ maintains 7 unpaired electrons, while Eu^3+^ does not. This the highest number of unpaired electrons in the series, which makes Gd^3+^ an ion with the largest spin angular momentum. However, the magnetic moment of an element also depends on the orbital angular momentum, which is zero for Gd^3+^. This lack of contribution from the orbital angular momentum brings the total magnetic moment of Gd^3+^ to the fifth place among lanthanides with the calculated and measured values of 7.94 and 7.90, respectively [6].

Electronic transitions in 4f orbitals also give rise to absorption and emission of Gd in the UV region of the spectrum, which are both fairly weak due to a low extinction coefficient that has been measured to be 4.20 M^−1^ cm^−1^ at 273 nm.

Like other lanthanides, Gd is a hard acid forming bonds that are highly ionic [7]. In aqueous environment, it most commonly exists in the 3+ oxidation state with an ionic radius of 0.99 Å. It is of great biological importance that the Gd^3+^ radius is similar to Ca^2+^ [7]. It has been reported that replacing Ca^2+^ with Gd^3+^ impacts essential biological processes including signaling [8]. It has also been noted that the trivalent ion demonstrated a higher binding capacity compared to the calcium ion [7]. The impact of lanthanides as inhibitors for cardiac and muscle cells were reported in the early 20th century as reviewed by Evans [9]. These experiments did not include Gd^3+^ although it was found to be biologically active as well, as discussed in Section 5 of this review.

In an aqueous environment, Gd^3+^ is known to be hydrated by 8–9 water molecules that form the inner sphere and reduce the positive charge on the trivalent ion by donating electrons. One of the important features of Gd^3+^ is its stronger affinity to oxygen as an electron donor. This property makes coordination with nitrogen or sulphur-containing ligands weak and inefficient. From the biological perspective, carboxylic groups on proteins and phosphate groups on nucleic acids are the primary source of oxygen for coordination with Gd^3+^. Other biologically relevant phosphates, oxides, and carbonates exhibit low solubility, thus, their interactions with Gd^3+^ in vivo may be neglected due to limiting bioavailability [10].

Gd has been reported to have seven stable isotopes and 30 radioisotopes [2]. In addition, it has displayed the ability to capture neutrons with high efficiency [2]. Quantitatively, this ability is reported as a cross sectional area expressed in units of barns, which is related to the probability that neutrons will encounter no interactions, be deflected, or be captured when passing through a given area [11]. Gd and some of its isotopes (Gd^155^ and Gd^157^, in particular) have extremely high cross-sections, even compared to elements used to control nuclear fission in reactors, which is attributed to the mass of a nucleus, i.e., the number of neutrons it contains [6]. This property of Gd enabled its use in shielding nuclear reactors.

Since Gd^3+^ does not serve any biological purpose, human exposure in the past was limited to consuming water contaminated with the metal leached from minerals [12] and inhaling aerosols released upon erosion [13]. The extent of exposure was insignificant since the ambient concentrations of Gd in nature remained at low ng/mL levels (e.g., [14]). It was not until the 1990s that the elevated levels of Gd in water due to anthropogenic activities were reported, naming Gd-containing compounds used as contrast enhancing agents in magnetic resonance imaging (MRI) one of the major culprits behind water contamination [14].

Upon entering the body, Gd^3+^ is distributed by the vascular system. Its deposition and long-term retention have been reported in the brain, kidney, liver and bones [15], as described in Section 4.2.

Putting together the risks of human exposure due to the elevated levels of Gd in nature, the fact that it may replace Ca^2+^ in the body and significantly impact biochemical processes at the cellular level, and, finally, the results of recent studies demonstrating accumulation and long-term retention of Gd^3+^ in the body, it seems appropriate to raise the question on the contribution of these factors to human health. In this review, the emerging environmental impact and potential alternatives to Gd^3+^ will be discussed, followed by some of the implications of Gd^3+^ interaction with biologically relevant molecules and cells. While a fair amount of work has been done on the impact of Gd^3+^ on ion channels, less is known about its effect on lipids and lipid organization within membranes. The latter topic seems particularly important considering the recently obtained data in the Prenner group indicates strong interaction of Gd^3+^ with biologically-relevant mammalian lipid classes. The data is presented in Section 5 of this review.

### Classification of Gadolinium Chelating Compounds

**Figure 1:**
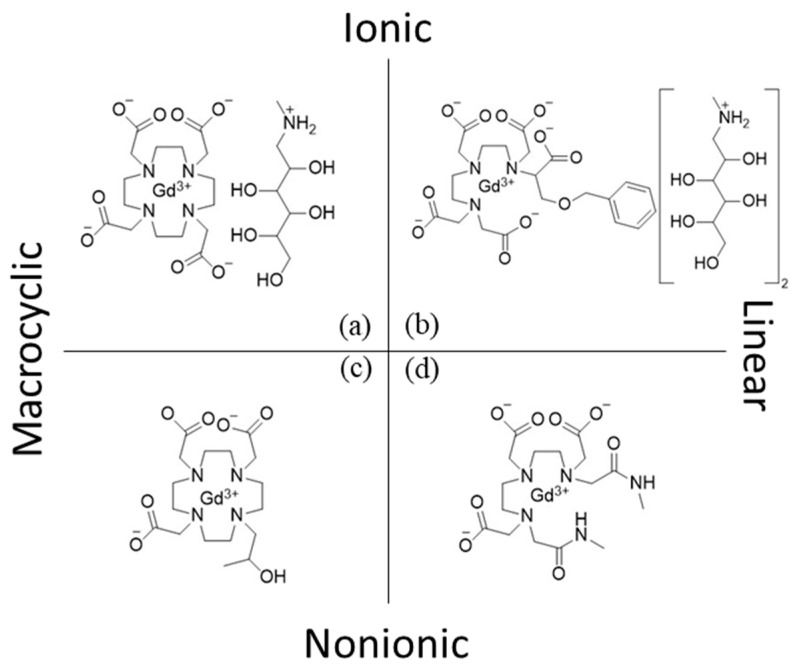
Structure and type of commercially approved gadolinium contrast agents used in MRI. (**a**) Gd-DOTA (Dotarem), (**b**) Gd-BOPTA (MultiHance), (**c**) Gd-HP-DO3A (ProHance), and (**d**) Gd-DTPA-BMA (Omniscan).

Gd^3+^ is known to bind to species with electron donating groups. In the body, such groups exist in proteins and nucleic acids. Therefore, administering Gd in its ionic form would lead to undesirable coordination with biomolecules and their building blocks. For example, phosphate groups are suitable candidates for forming non-soluble complexes. Moreover, free Gd^3+^ is known to cause detrimental effects at the cellular level and lead to debilitating diseases such as nephrogenic systemic fibrosis (NSF) [15]. In order to minimize side effects and increase the blood circulation time of Gd^3+^ for the use in biological applications, it is usually administered in complex with chelating ligands. Gd chelating compounds are either linear or macrocyclic species. Their structure affects the thermodynamics and kinetics of complexation. Figure 1, seen below, provides an overview of compounds that were used to design various chelate classes. The interested reader is referred to Ebrahimi et al., which provides details on the clinical use of these compounds and their thermodynamic stability [16].

Overall, the formation of Gd-ligand complexes is a thermodynamically favorable process both with respect to changes in entropy and enthalpy. The interaction of a hydrated Gd^3+^ ion with any octadentate ligand leads to a release of eight out of nine inner sphere water molecules, which increases entropy. In addition, electro static interaction between the Gd^3+^ ion and negatively-charged groups on the ligand results in a favourable enthalpic contribution to the overall process.

In the context of thermodynamics, one important point to make is that nitrogen containing chelating groups in the macrocyclic ligands are more basic compared to the corresponding groups in the linear ligands. Naturally, higher basicity implies that the group is more willing to donate a lone pair of electrons. Thus, the bonds formed between more basic groups and the metal centre are stronger. As a consequence, Gd^3+^ complexes with macrocyclic ligands, in general, are expected to be more thermodynamically stable.

It is tempting to characterize the stability of these complexes using an equation for the binding constant that is well known from thermodynamics texts:

(1) $$K=\frac{\left[Gd\ldots L\right]}{\left[Gd\right]\left[L\right]}$$

where the concentrations on the right-hand side represent the equilibrium concentrations of the complex, free metal ion, and free ligand. In this instance, the entire analysis is reduced to calculating a single number. However, this simplistic approach often fails in practice as it neglects factors that become relevant under certain experimental conditions. In particular, it assumes that all ligand molecules are deprotonated. This assumption does not hold true at the physiological pH, at which protons compete with Gd^3+^ ions for lone pairs of electrons on the ligand. To account for the fact that some sites on the ligand molecules are protonated, the equation for the binding constant may be modified as:

(2) $$K_{conditional}=\frac{K}{\alpha_{HL}}$$

where $\alpha_{HL}$ is a coefficient that reduces the binding constant due to the ligand protonation. It is defined as:

(3) $$\alpha_{HL}=1+\left[H^{+}\right]K_{1}+{\left[H^{+}\right]}^{2}K_{1}K_{2}+\ldots +{\left[H^{+}\right]}^{n}K_{1}K_{2}\ldots K_{n}$$

The concentration of $\left[H^{+}\right]$ is defined by the pH of the solution, and the constants $K_{1},K_{2},\ldots K_{n}$ represent the stepwise protonation constants of the ligand [17,18].

**Figure 2:**
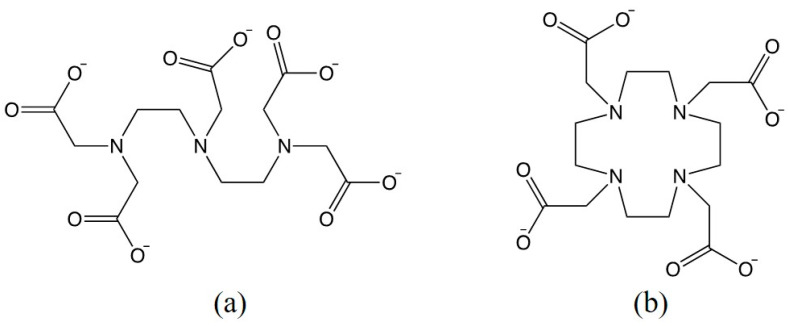
Structures of DTPA (**a**) and DOTA (**b**).

A reduction in the stability constant may be illustrated using two ligands known as diethylenetriaminepentaacetic acid (DTPA) and dodecanetetraacetic acid (DOTA), which are both FDA approved compounds for the use in MRI [19]. Their corresponding structures are shown in Figure 2. As a linear amine, DTPA is less basic compared to cyclic DOTA. The binding constant calculated without accounting for protonation at the physiological pH is higher for DOTA. However, higher basicity of the amine groups in DOTA also implies stronger competition between Gd^3+^ ions and protons. As a result, $K_{conditional}=0.75\times K$ for DOTA at the physiological conditions of pH 7.4. In the case of DTPA, $K_{conditional}$ is also reduced compared to K at pH 7.4 but the reduction is to a lesser extent ($K_{conditional}=0.8\times K$). While the relative reduction is greater for DOTA, the absolute values of $K_{conditional}$ indicate that macrocyclic chelates provide higher thermodynamic stability than the linear ones [20]. An interested reader is referred to Port et al., for a full list of stability constants for currently marketed Gd-chelates [20].

Upon intravenous injection, this polar complex rapidly distributes within the extracellular matrix because it cannot cross cell membranes [21]. Several studies report that there is little binding to plasma proteins and almost complete excretion via the kidneys [22], although Gd^3+^ accumulation has been shown as discussed in Section 4.

**Figure 3:**
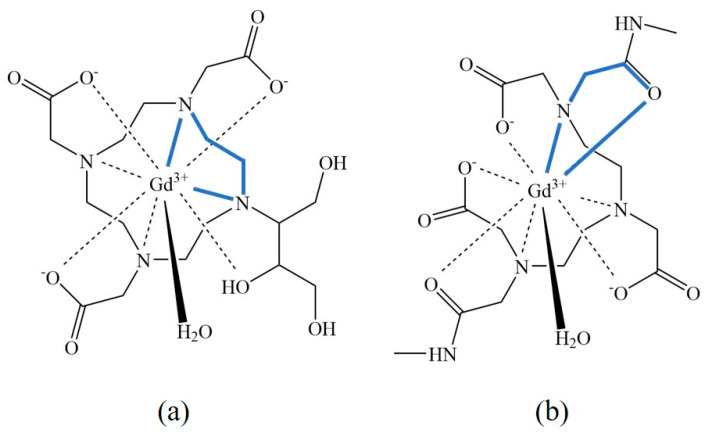
Structure of gadolinium chelates (**a**) Gadovist and (**b**) Omniscan, highlighting the two types of five-membered-rings formed between gadolinium with nitrogen and oxygen.

All currently clinically used Gd-chelates are based on octadentate polyaminocarboxylate ligands. Considering that Gd^3+^ has a coordination number of 9, one free site remains available for coordination with a water molecule. Coordination with donor atoms within the ligand results in the formation of five-membered rings. Not to be confused with five-membered rings in cyclic compounds like furan, the rings found in chelates are formed due to the formation of ionic bonds between nitrogen and carboxylate donor atoms and the central ion (Figure 3) [23,24].

A higher number of five-membered rings leads to lower steric strain in the chelate [25]. Linear Gd^3+^ ligands are capable of forming seven five-membered rings, while macrocyclic ligands may form up to eight rings with the central atom and create structures that are viewed as more thermodynamically stable [20]. From the geometrical perspective, macrocyclic chelates are also more suited for chelation of Gd^3+^ as they feature an internal cavity with oxygen and nitrogen binding sites capable wrapping around Gd^3+^ ions [20].

In vivo, the kinetic stability of the complexes needs to be taken into account. Dissociation of Gd^3+^ from the chelating molecules is described by either decomplexation kinetics, corresponding to spontaneous or proton-assisted dissociation, or transmetallation kinetics in, which Gd^3+^ is displaced by endogenous metals, e.g., Ca^2+^ and Zn^2+^ [20,26]. At physiological pH 7.4, the contribution of proton-assisted dissociation to Gd^3+^ leakage is thought to be negligible as the concentration of H^+^ is very low and total excretion of the complexes is assumed before any harmful amount of Gd^3+^ leakage occurs [27]. The same effect was studied under acidic conditions (pH 1.0) which confirmed the higher stability of macrocyclic chelates over open-chained chelates. The dissociation half-life for various macrocyclic chelates under this condition, spanned hours to days, while the half-life for open-chained chelates was on the order of seconds [20]. The presence of Ca^2+^ and Zn^2+^ also led to the formation of complexes in which these ions displaced Gd^3+^ in some of the chelates, the notable exception being the ionic macrocyclic chelate DOTA [20].

## 2. Applications of Gadolinium

The element has found use in a broad range of highly technological applications. Its consumption continues to grow resulting in potentially harmful impact on ecological systems (Section 3) and human health (Section 4).

### 2.1. General Technological Applications of Gadolinium

Gd is a silvery metal that is readily workable and ductile. Its addition to other metals in amounts as small as 1% significantly improves the malleability and resistance to oxidation [28]. The benefits of spiking metal alloys with Gd is particularly pronounced in case of manganese that has low mass and needs protection against oxidation [29].

Gd is used to improve the working characteristics of the synthetic yttrium iron garnet ceramics that found applications in sensors, lasers and phosphorescence emitters in the microwave frequency range (Lamastra et al., and references therein [30]). The addition of Gd extends garnet applications to wider temperature and power values [31]. Colbalt and Gd were employed in the development of the rewritable magneto optical disk by IBM [32].

For an overview of applications of Gd an interested reader is referred to Rim et al. [33]. At this point, it feels appropriate to mention that the increasing industrial demand and use of Gd resulted in a substantial environmental impact that has been documented (e.g., Rogowska et al. [2]). More discussion can be found in Section 3.

The main applications that are within the scope of this review are biomedical applications as described below.

### 2.2. Biomedical Application of Gadolinium

The magnetic properties of Gd^3+^ have cemented its role as an essential component of diagnostic MRI. To understand how the properties of Gd^3+^ aid in improving the quality and resolution capabilities of MRI, the fundamentals of the technique must be understood.

MRI is based on nuclear magnetic resonance that is observed in atomic nuclei possessing a magnetic moment. The magnetic moment only arises in nuclei with a non-zero spin angular momentum. Spin is a purely quantum mechanical concept. One may crudely think that the spin emerges due to charges in the nucleus producing “current loops”. Classically, such moving charges generate magnetic fields. In some atomic nuclei, the magnetic moments cancel out, which makes them silent in MRI. On the other hand, hydrogen, an element abundant in the human body, is an example of an atom that has a nucleus with a non-zero magnetic moment.

**Figure 4:**
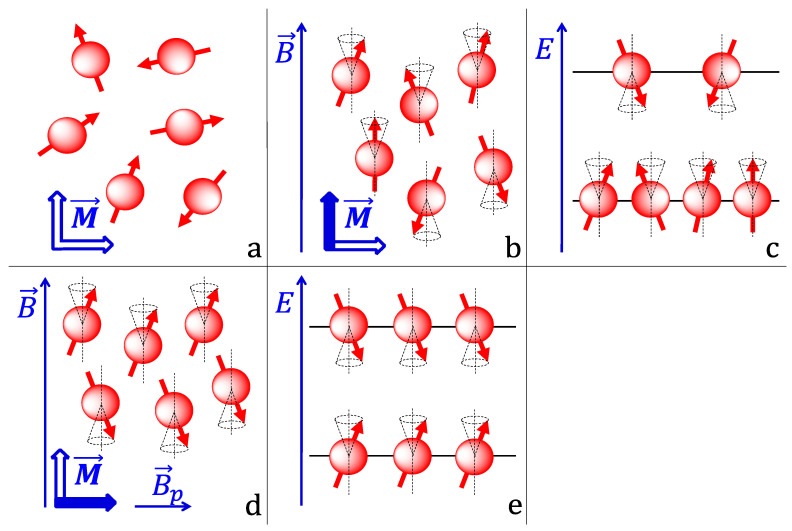
Depiction of spin and orientation of hydrogen nuclei under a magnet. (**a**) Nuclei displaying random orientation in the absence of a magnetic field. (**b**) Nuclei aligned along or against the magnetic field lines. (**d**) Nuclei aligned in equal amounts along and against magnetic field lines. (**c**,**e**) Nuclei separated into energy levels based on alignment.

Naturally, the orientation of the magnetic moments in different hydrogen nuclei is random (Figure 4a). This results in no net magnetization in any direction because the magnetic moments cancel each other out (zero magnetization is schematically depicted in Figure 4a as two blue empty arrows). However, when a human body is placed in a strong static magnetic field, the magnetic moments couple with it and align themselves either along or against the field direction (Figure 4b). Such alignment is forced upon the magnetic moments because quantum mechanically there exists only two energy levels available for the nuclei to occupy under these conditions. These energy states correspond to two different orientations—the nuclei with the magnetic moments oriented along the field have less energy compared to the nuclei with the magnetic moments aligned against it. According to the Boltzmann distribution, at thermal equilibrium the number of nuclei in the lower energy state is higher compared to the number of nuclei occupying a higher energy state (the effect is highly exaggerated in Figure 4c; in reality the occupation numbers for two states are marginally different). This population imbalance, however, is the root cause of a net magnetization that emerges in the direction of the external magnetic field (shown as a blue solid arrow in Figure 4b) and whose magnitude is proportional to the population difference. The origin of the net magnetization may become more intuitive if one adds the projections of all vectors representing individual magnetic moments onto a vertical axis (Figure 4b). It is clear that the larger the population difference is, the more vector components point upwards, the larger the sum of these components is.

The magnetic moments are not perfectly parallel or anti-parallel to the field. They are tilted at a small angle (Figure 4b). The field exerts a torque on the magnetic moments causing them to precess on a cone with a so-called Larmor frequency that is directly proportional to the field strength. Since they precess out of phase (at a given moment of time their positions on the imaginary cones are random), there is no net magnetization in the plane perpendicular to the direction of the field because the projections of the magnetic moments onto that plane cancel each other out (schematically depicted by the blue empty arrow in Figure 4b).

In a typical MRI procedure, the net vertical magnetization is first removed by applying a short (usually several microseconds) radiofrequency pulse that has the magnetic vector rotating at the Larmor frequency in a plane perpendicular to the external magnetic field. To gain a better understanding of the process, it is constructive to follow the net magnetization vector from a different frame of reference, namely the one rotating with the magnetic vector of the applied pulse. Since this new frame of reference rotates at the same frequency as the individual magnetic moments, no precession is observed, and the magnetic moments appear stationary. Therefore, only the effects from the magnetic field of the pulse should be considered, which are two-fold. Since the difference between the energy states corresponding to the magnetic moments aligned along and against the external field matches the energy of a photon from the applied radiofrequency pulse, resonance is observed, i.e., individual nuclei absorb photons from the pulse and transition from the lower to the higher energy state. Naturally, this process leads to a decrease in the population imbalance—the number of magnetic moments in the lower energy state goes down and the number of magnetic moments in the higher energy state goes up. When the population numbers become the same (Figure 4e), the vertical components of the magnetic moments cancel out and the net vertical magnetization disappears (schematically depicted as the blue empty arrow in Figure 4d). Additionally, the pulse brings the individual magnetic moments into phase, i.e., after the pulse has passed, the vectors point into the same direction on the corresponding cones (Figure 4d). Synchronization of the phases results in the appearance of the net magnetization in the horizontal plane. Indeed, adding all the vectors shown in Figure 4d lead to a net horizontal component.

The processes leading to restoring the thermal equilibrium are called relaxation. The spins may lose energy and return to a lower energy state, restoring the population imbalance and net magnetization along the field. This process is known as spin-lattice relaxation because the energy is lost to the surroundings and it has a characteristic time conventionally denoted T_1_. A return to the state of thermal equilibrium is also accompanied by a loss of magnetization in the plane perpendicular to the field. This process requires dephasing of the magnetic moments and is referred to as spin-spin relaxation. In this process, the precession of individual magnetic moments is affected by local magnetic fields due to neighboring magnetic moments. This interaction leads to dephasing with the characteristic time denoted T_2_. In general, T_1_ is about an order of magnitude slower than T_2_.

A strength of the signal in MRI is determined by the magnitude of the net magnetization vector created by the radiofrequency pulse. Tissues, in which this vector is large, appear white in the images. On the contrary, tissues with a weak response to the radiofrequency pulse, appear dark. Furthermore, important data may be obtained by collecting T_1_ and T_2_ weighted images separately. Typically, water and lipid molecules represent two extreme cases with respect to the decay times. The differences arise due to structural peculiarities of the molecules. Hydrogen atoms in water are linked to an electronegative oxygen which withdraws electrons leaving the hydrogen nuclei less shielded and, therefore, more susceptible to the external magnetic field. Furthermore, water is a small molecule with a wide range of vibrational frequencies. As a result, only a small fraction of water hydrogen nuclei may effectively transfer energy to the molecular lattice, leading to long T_1_ relaxation times for water. On the contrary, hydrogen atoms in lipids are connected to electroneutral carbon atoms that do not deplete their electron density. When placed in an external magnetic field, these electrons act as a shield. Lipid molecules in tissues are tightly packed; their bonds vibrate at frequencies close to the Larmor frequency of the magnetic moments, thus, making the process of losing energy to the molecular lattice effective, which manifests itself in short T_1_ relaxation times.

Contrast in MRI images may be improved by enhancement agents. These compounds do not appear in the images but rather affect the relaxation times T_1_ or T_2_. Gd^3+^ belongs to a group of so-called T_1_ contrast enhancement agents as it is known to reduce the T_1_ relaxation time of water. All Gd^3+^ complexes approved for use in diagnostic imaging are nine-coordinate complexes in which a ligand occupies eight binding sites at the metal center and the ninth coordination site is occupied by water molecules. It is the magnetic moment in the Gd^3+^ centre that induces magnetic relaxation in the hydrogen nucleus of the coordinated water molecule and shortens the T_1_ relaxation time of its proton. Relaxivity is determined by various structural parameters including rotational correlation time, electronic relaxation time, water residence lifetime, and the distance between Gd^3+^ and the coordinated water hydrogen [34].

As a trivalent metal ion with the capacity to interfere with many biological systems, Gd^3+^ cannot be administered on its own. Instead, it must be complexed with a suitable ligand that prevents unwanted biological interactions and allows Gd^3+^ to be excreted from the body following administration. While the majority of Gd^3+^ complexes used for MRI are stable enough to persist until they are naturally flushed out by the body, Gd^3+^ dissociation is still an issue of concern and efforts have been made to produce more efficient contrast agents that can also dual-function in MRI and therapeutics. These have been developed in the form of Gd-containing microspheres and Gd-labelled nanoliposomes [35,36]. The former can be made of a copolymer of Gd-methacrylate and methacrylic acid with Gd^3+^ loaded via electrostatic interactions with three carboxylic acids [35]. The applicability of these were studied by performing MRI scans on tumor bearing mice over 4 h. The microspheres were demonstrated to be strong contrasting agents, as their presence enhanced the signal of T_1_-weighted MRI images [35]. Furthermore, the specific longitudinal relaxivity was evaluated and found to be more than double the value of the open-chain contrast agent Magnevist, further showing its usefulness in diagnostic imaging. The use of nanoscale contrast agents also has advantages over traditional ones, as nanocarriers can be loaded with hundreds of thousands of Gd^3+^ atoms, reducing the amount of contrast agent required [36]. Additionally, the carrier can be coated with ligands such as polyethylene glycol (PEG) and polysaccharides to assist in passive or active targeting and increase circulation time [36]. In Gd-labelled nanoliposomes, Gd^3+^ is directly chelated to the head group of 1,2-distearoyl-sn-glycero-3-phosphoethanolamine-*N*-diethylenetriaminepentaacetic acid (PE-DTPA) [36]. This is advantageous as Gd^3+^ is localized in the lipid bilayer and can readily interact with water molecules, necessary for enhanced T_1_ relaxivity [36]. Gd-DTPA has also been incorporated into other nanocarriers, including block copolymers and PEG-b-polyaspartic acid doped calcium phosphate nanoparticles, both of which showed significantly higher relaxivity than small-molecule Gd-DTPA [37].

Along with diagnostic imaging, MRI has found use in studying cellular processes at the molecular and genetic level. In order to increase the viability of intracellular imaging using MRI, the contrast agent Gd(DOTA) can be coupled to a cell-penetrating peptide and a specific peptide nucleic acid. To demonstrate this idea, Mishra et al., formed the complex with a peptide nucleic acid anti-sense to the mRNA of DsRED2, a red fluorescent protein [38]. CCL-11 cells, which were genetically altered to express DsRED2, were used to determine uptake and binding capabilities of the complex. Successful binding to the desired mRNA target was indicated by significantly altered T_1_ relaxation times in these cells, with relaxivity values comparable to that of traditional Gd-DOTA. This showed the ability for cell penetration and targeted imaging of cellular processes [38,39]. Cellular uptake and affinity for certain tissues was demonstrated using fluorescence imaging of the complex labelled with the fluorescent dye fluorescein. The lipophilic nature of the complex increases its abundance in liver and kidney tissue while suppressing its ability to cross the blood-brain-barrier (BBB). This approach demonstrates a viable method for intracellular imaging using MRI, with the potential to target any biological molecule of interest.

Recently, Gd-based nanoplatforms that mimic red blood cells have been proposed as T_1_ contrast enhancement agents. This approach takes advantage of the biocompatibility and in vivo longevity of red blood cells that could outperform current Gd based contrast agents (GBCA) [40].

### 2.3. Therapeutic Applications

In addition to utilizing Gd^3+^ in MRI imaging and diagnostics, it also found its application in therapeutics. It has been established that liposomal ^157^Gd^3+^ formulations represent a promising approach for neutron capture therapy of glioblastoma cells. Neutron capture therapy is carried out by first loading cancerous cells with ^157^Gd^3+^ which has a high propensity to capture thermal neutrons. The cells are then radiated with neutrons, acquired from the source, which is either a nuclear reactor or an accelerator. After losing energy as they penetrate tissue, the neutrons are captured by ^157^Gd^3+^, which subsequently emits high-energy alpha particles that kill adjacent cells. Obtaining a sufficient Gd^3+^ concentration in cancer cells is a key factor in neutron capture therapy. In a study by Peters et al., composite liposomes were used as a drug carrier to deliver ^157^Gd-chelates into cancer cells [41]. Liposome composition was found to drastically change the effectiveness of this technique due to varying endocytic pathways and ^157^Gd^3+^ cellular uptake. The most effective liposome compositions for targeting ^157^Gd-chelates into cancer cells were 1,2-dioleoyl-sn-glycero-3-phosphocholine (DOPC): cholesterol: 1,2-dioleoyl-3-trimethylammonium-propane (DOTAP) with a molar ratio of 57.41:33.35:9.23, respectively and DOPC: cholesterol: 1,2-dioleoyl-sn-glycero-3-phosphoethanolamine (DOPE) with a molar ratio of 70:20:10, respectively. Liposomes composed of cardiolipin did not prove effective in delivering ^157^Gd-chelates into cancer cells. Importantly, ^157^Gd^3+^-loaded liposomes have a dual purpose. On one hand, they may be used as an MRI contrast agent for enhanced diagnostic and surveillance purposes. On the other hand, they act as a therapeutic agent that destroys cancel cells, thus enabling a theranostic approach [41].

Gd^3+^-based drugs have been considered for therapeutics since they are naturally cytotoxic due to their ability to reduce cell proliferation and induce apoptosis [42,43,44,45]. These effects are particularly useful against cancerous cells that do not respond to apoptosis stimuli, and as a result, proliferate uncontrollably. A study on HeLa cells, an immortal cervical cancer cell line, conducted by Long et al., found a GdCl_3_ concentration-dependent response to cell proliferation [46]. At low concentrations of GdCl_3_ (≤0.5 mM), there was a reported increase in cell growth after 24 and 48 h, with the highest growth percentage of 22.4 ± 1.27%, occurring at a concentration of 0.01 mM. At higher concentrations (≥1.0 mM), growth suppression occurred. When cells were cultured with 2 mM GdCl_3_, proliferation was suppressed by 26.4 ± 1.03% after 24 h, and 64.0 ± 2.68% after 48 h. In order to elucidate the mechanism behind these results, the profile changes of extracellular metabolites were examined using high-throughput methods, such as liquid chromatography coupled to linear ion trap (LC-LTQ) and ion trap time-of-flight mass spectrometry (IT TOF MS). The metabolite profile gives insight into the cellular state and metabolic processes that were triggered as a result of GdCl_3_ treatment. A total of 48 metabolites were identified and grouped into functional categories. It was found that at lower GdCl_3_ concentrations, lipid metabolism-related molecules, such as lauryl-CoA, malonylcarnitine, and phosphatidylinositol were expressed in higher amounts. These are involved in fatty acid elongation, transport, energy production, and signal transduction, necessary processes in proliferating cells [47,48,49]. Other enhanced metabolites were precursors for synthesis of nucleic acids, proteins, and lipids that are important for cell division. In HeLa cells incubated with high GdCl_3_ concentrations (2.0 mM), a high degree of down-regulation occurred, namely in metabolite precursors for amino acid synthesis, glycolysis, and ATP synthesis. The inhibitory effects on glycolysis are of special interest, as tumor cells over-express enzymes associated with glycolysis, such as various dehydrogenases, enolases, and aldolases, enabling cell proliferation under anaerobic conditions [50,51]. Therefore, glycolytic enzymes are important targets for cancer drugs, and the data presented in this study suggests that GdCl_3_ inhibits some of these enzymes. Moreover, metabolites in the glutathione and redox metabolic pathways were also decreased at higher concentrations of GdCl_3_, indicating the importance of these processes in cell growth. Other studies have shown that the antioxidative system and redox homeostasis are necessary for cell proliferation [52,53]. Motexafin gadolinium (MGd) has demonstrated therapeutic potential by disrupting these pathways via targeting thioredoxin reductase and ribonucleotide reductase, which was manifested in reduced amounts of key metabolites such as glutathione [54]. The results of that study showed that 20 µM MGd induced oxidative stress, by generating reactive oxygen species (ROS) in the presence of NADPH and oxygen. MGd also strongly inhibited ribonucleotide reductase, with an IC_50_ of 2 µM, preventing DNA synthesis and repair, and ultimately cell growth and proliferation.

Another important effect of GdCl_3_ on cells is the induction of apoptosis. A study by Ye et al., revealed that apoptosis on liver cell line 7701 was initiated through the mitochondria, and that ROS may be part of the mechanism [55]. In another study by the same group, the apoptotic signaling pathway and role of ROS were further elucidated by treating human embryo liver L02 cells with GdCl_3_ [55]. GdCl_3_ concentrations of 200 µM increased ROS levels after 12 h. As apoptosis was observed after 24 h, this elevation in ROS may be an upstream event of Gd-induced apoptosis. The accumulation of ROS decreased mitochondria membrane potential, inhibiting functionality. Moreover, DNA fragmentation and increased membrane permeability were observed, as indicated by propidium iodide staining. Large-scale DNA breakage stimulates the DNA-repairing factor, poly (ADP-ribose) polymerase [56]. Ultimately, all these factors resulted in increased intracellular levels of apoptosis-inducing factor, an initiator of the caspase 3 independent cell death pathway [57]. The addition of antioxidants *N*-acetylcysteine and dimethylthiourea, inhibited GdCl_3_-induced apoptosis. This further supported the role of ROS in cell death, as these two antioxidants remove intracellular ROS.

Another method of combating cancerous cells with Gd^3+^ is through photo activation therapy. This approach has the advantage of being able to tune the X-ray excitation energy to target electrons in the K-shell of high atomic number elements like Gd. Using X-rays within a narrow frequency range prevents damage to other tissues. The exposure causes ejection of inner K shell electrons. The formed vacancies are subsequently filled by electrons from higher energy levels. The energy released as a result of this jump may be absorbed by other electrons, which can subsequently escape the atoms. These electrons, known as Auger electrons, have been shown to induce DNA damage and cell death [58,59]. A study by Matsumoto et al. [60], used mesoporous silica nanoparticles (MSN) loaded with Gd^3+^ to target tumour masses from ovarian cancer cells and mark them for X-ray irradiation. Upon cellular uptake by endosomes, the Gd-MSN are localized in lysosomes close to the nucleus, where Auger electrons have the potential to do the most damage to DNA. Near complete destruction of tumour spheroids was achieved with 20 ng of Gd-MSN at an X-ray energy of 50.25 keV. These results show the potential for targeted therapy with minimal side effects from either Gd^3+^ or high energy X-rays.

## 3. Gd in the Environment

Gd has found use in many different industries (see Section 2.1 for examples). Old electronics and other waste that end up in landfills are potential sources of contamination that may occur through leaching into the environment over time. Furthermore, as discussed in Section 3.1, Gd content is substantially higher in soil and plants close to the mining and processing sites. However, the main environmental concern with respect to Gd is related to natural waterways and drinking water infrastructure.

An early report on excess of Gd in water was presented in 1996 showing an alarmingly increased concentration of 18 ng/L over the background level of just 0.54 ng/mL for the city of Berlin [14]. These values progressively increase over time correlating with the onset of Gd^3+^ use in MRI diagnostics, particularly following the approval of Gd-DTAB as a contrast enhancing agent in 1988 [61]. By now, Gd is found in river water, tap water, ground water, and seawater. It is detected at elevated concentrations at the effluents of wastewater treatment plants ([16,62] and references therein). So far Gd is considered a microcontaminant of emerging concern, i.e., a compound that can be analytically detected at low concentrations but lack sufficient toxicity data and regulatory requirements to monitor levels [16]. In general, it is difficult to establish universal safe levels due to large variations in the natural background.

Determination of Gd concentration is usually done as part of analysis for lanthanides. The elements exhibit similar chemical behavior (see Section 1) and occur in mixtures in nature. However, their levels vary with the area geology. Therefore, Gd concentrations determined experimentally need to be corrected for the geological background. Several standards have been used in the literature as described by Ebrahimi and Barbieri [16,63], however, the post-Archean Australian shale (PAAS) remains the most widely used standard composite, as it represents the best average of the Earth’s crust [64].

To improve the data accuracy on the geological background, the concentration of rare earth elements (in some cases including yttrium) are normalized to the PAAS distribution to determine the actual geological background as Gd_Geo_/Gd_PAAS_. This value is then subtracted from the total measured Gd content to calculate the anthropogenic contribution.

In addition to elevated Gd levels reported for Berlin, an analysis of 20 Dutch water samples was performed and no clear correlation between Gd content with pH, nitrate or carbonate was observed. Gd values also varied with the depth of sampling, from aquifers to surface water. The authors suggested a value of 10 μg/mL Gd as an admissible drinking water concentration. This number was derived from toxicity studies on rats with a factor of 1000 to account for intra- and inter-species variation and extrapolation to chronic exposure [65].

There is a strong correlation between Gd concentration in the environment and the level of the health care system [61] as Gd is found in excessive amounts closer to larger cities and hospitals performing MRI. Concentrations vary with the population density and proximity to wastewater plants. It is instructive to analyze, for instance, the data obtained for a hospital in Freiburg, Germany. The Gd content in the hospital effluent was measured to be low in the morning. However, this number kept increasing during the day and peaked in the afternoon, consistent with the treatment schedule and expected excretion of the 1.1 g Gd dose that is typically administered into a 70 kg adult patient in the hospital. Values of 7 μg/L were reported 39 days after treatment, which could explain the lower Gd reading for smaller cities and rural areas due to outpatients [66]. Another correlation of Gd level linked to the use of MRI is the report that the current pandemic of COVID-19 reduced Gd levels due to an 80% reduction of tests in order to allow for more intensive care capacity [61].

The thermodynamic stability of Gd chelates varies and depends on the structure of the chelating agent [61]. The first approved and initially widely used linear Gd-DTPA (Magnevist) contrast enhancement agent was among 4 linear GBCAs that were recommended to be abolished by the European Medicines agency in 2017 [61]. Macrocyclic GBCAs are more stable but a closer examination showed that they and other GBCAs are not removed in wastewater treatment plants and end up in rivers with much higher concentrations in densely populated and industrial areas [15]. Thus, Gd content in rivers flowing in industrialized areas of Brazil and South Korea is higher compared to less populated regions of the same countries [16].

Another important source of Gd contamination is the wastewater released from sewage pipes into the oceans as documented for the Atlantic Ocean off the coast of Brazil as well as for Kona coast at the Big Island of Hawaii ([2,16] and references therein). A detailed study looked into the use of the 6 km long submarine sewer outlet into the Atlantic Ocean from the city of Salvador the Bahia in Brazil. The analysis of wind and current patterns in the region confirmed the pollution of the coast, including beaches used for recreational purposes [67].

The growing concentrations of Gd in the environment inevitably raise the question of its ecological impact, which is addressed in the next section.

### 3.1. Toxicity and Ecological Impact of Gd

Toxicity studies of Gd in nature are scarce but there are a few examples indicating potential danger.

Limited toxicity of Gd compounds for *Pseudomonas putida* was reported as a 10% reduction in population upon exposure to 870 mg/L [66]. Additionally, some algae were found to be resistant and the no-observed-effect-concentration used in European risk assessment studies was determined to be 100,000 ng/mL [2].

The more complex single cell protozoa *Tetrahymena shanghaiensis* was exposed to rare earth metals Gd, La, Sm, and Y. Gd concentrations were ~2× (Y), ~4× (Sm), and 6× (La) lower than these other lanthanides but ~30× higher than that of Cd, which was used as positive control [68].

On a higher level of complexity, sea urchin embryos are used in developmental biology and also as tools to assess ecotoxicology [16,69]. Since Gd severely affected development of the embryos and skeleton growth of the sea urchins, the importance of Gd toxicity tests on several species for risk assessment was highlighted [16].

A comparison of two European and two Australian sea urchin species showed that values for 50% inhibition varied greatly between 56 nM and 132 mM. The most striking result were major alterations or inhibitions of skeleton growth within 48 h. A similar phenotypic response of skeletal impact was reported for three Japanese species [70], suggesting a conserved mechanism.

One important observation was that Gd^3+^ affected calcification, presumably because Gd^3+^ competes with Ca^2+^. Such effects are especially relevant since many marine species have calcifying larvae stages and, thus, Gd^3+^ pollution may be even more alarming as it could impact the food chain in the oceans [71].

Another important aspect of ecosystem complexity is communication between different organisms. For bacterial cultures this is represented by the term quorum sensing [72], i.e., the ability of bacterial strains to release and receive molecules as signals to adapt to changes in the living conditions. In order to enable the broad scale screening of potential toxic agents or toxic mixtures, bioassays that allow simple and quick testing are required. They have been developed for many organisms from bacteria, to algae, mollusks, or fish [72].

A particularly suitable example was commercialized as MicroTox TM and is using cultures of Aliivibrio fisheri that emit blue-green luminescence measurable under high throughput conditions [73]. This assay was used to compare the toxicity of 4 critical toxic agents. A 20% population reduction was induced in the following order of decreasing toxicity: Cu^2+^ (3.2 mg/L) < nanoAg (3.4 mg/L, reported) < Gd^3+^ (34 mg/L) < microplastics (2.6 g/L). In addition, copper toxicity was observed to increase in the presence of the other agents, thus, synergistic toxic effects need to be considered [72].

Another interesting example is a ternary culture that included the algae *Euglena gracilis Z*, as a food supplier, the protozoa *Tetrahymena thermophila B* as a user, and bacteria *Escherichia coli DH5a* to provide metabolic breakdown [74] whereby each species benefits from the others. These systems can be cultured for extended periods of time, as long as 12 months. Gd^3+^ was first added after 56 days to ensure that the mixed culture was well established. Upon addition of 300 μM Gd^3+^, *E. coli* died out shortly after metal addition, whereas the protozoa were reduced temporarily and recovered after 87 days. The algae were not affected, while same concentration was toxic to all components individually. A higher concentration of 1000 μM was required to eliminate the whole community [74].

Gd^3+^ toxicity based on the ability to block Ca^2+^ channels in a range of 1–200 mM was shown for sea urchins, *Xenopus* oocytes and mammalian cell lines. Furthermore, Gd^3+^ at a concentration of 250 mg/L decreased the viability of rainbow trout hepatocytes within 24 h (for details see [71] and references therein).

Overall, this brief summary clearly demonstrates a significant toxicity impact that Gd^3+^ exerts across the entire spectrum of life, from bacteria to humans.

The next section will address remediation efforts in wastewater treatment and options to avoid the release of Gd^3+^ into the water ways in the first place, including efforts to replace Gd^3+^ for imaging.

### 3.2. Remediation and Alternatives

Wastewater treatment processes will not be discussed in detail as this discussion is beyond the scope of this review, and only a brief description will be provided. In general, wastewater treatment is performed in three stages [75]. The first stage includes sedimentation of solids in holding tanks, while floating elements (e.g., fats), are collected from the top. In the second stage, the waste products are broken down by bacterial cultures. The third stage may consist of passing the water through multiple filters and storage in lagoons to allow further microbial breakdown [75].

The standard analysis of wastewater includes classical chemical parameters such as the chemical oxygen demand, the biochemical oxygen demand, and the amount of total dissolved solids. While these are relatively easy and fast tests, they do not provide any information on the impact that the treated water may have on living organisms. Therefore, additional bioassays have been developed as important tools ([76] and references therein).

One key model system are cultures of Daphnia, which belong to the planktonic crustaceans. Indeed, *Daphnia magna straus* is widely used because it can be grown easily and fast in a laboratory. More importantly, it is highly sensitive to many pollutants [76,77] and provides an excellent tool to assess the performance of a wastewater treatment plants in terms of toxicity.

While naturally occurring lanthanides are mostly found in sludge, organic GBCA end up in the water phase, and this release leads to the increased concentrations of Gd^3+^ over other lanthanides, termed the Gd^3+^ anomaly in water [62]. It was also demonstrated that GBCA, in contrast to Gd^3+^, have low adsorption affinity during water filtrations steps [61,78].

Another important point for consideration in treatment of wastewater containing GBCA is a step in which ferric or aluminum coagulants are added to induce sedimentation of chemicals and colloids [61]. The stability of Fe^3+^-EDTA complex is much higher than Gd^3+^-EDTA complex [66]. This allows for the process of transmetallation to occur, which further challenges the water treatment plants [79] because EDTA gets stripped from the Gd^3+^ ion in the presence of iron, and a bare Gd^3+^ that is substantially more toxic compared to its complex becomes released ([66] and references within).

The standard toxicity test for pollutants such as Gd on Daphnia represents a series of dilution experiments aimed at determining the pollutant concentration at which all daphnia are able to swim.

Some reports show progressive removal of Gd from primary to secondary to tertiary treatment at 29%, 76% and 100%, from a treatment plant in India [76]. In contrast, studies form large wastewater plants in the US indicate the persistence of anthropogenic Gd. Much improved removal was achieved by reverse osmosis included in a so-called advanced water treatment plant in Queensland, Australia [80]. An interested reader is referred to [16], and references therein, for more information on a variety of wastewater treatment processes.

Other approaches to Gd removal from wastewater include chemical oxidation, reactions involving radicals, and photochemical and electrochemical processes [81].

One easy solution aimed at eliminating Gd release into the sewer involves collecting patient urine for 24 h [61]. A recent report from Germany documented high patient compliance with this approach [82]. Any process that could help to reduce or prevent the release of Gd into aquatic systems would be extremely impactful considering that 19 and 21 tons of Gd are released into the environment per year in the EU and the USA, respectively [61]. All these considerations, clinical and ecological concerns, led to significant research efforts for viable alternative for GBCAs for imaging purposes. Paramagnetic compounds with a large number of unpaired electrons are desirable as T_1_ contrast agents. Thus, aside from Gd^3+^, Mn^2+^ and Fe^3+^ are prime candidates [83].

A variety of scaffolds, from liposomes, to dendrimers and nanotubes, to name a few, were explored [84,85,86,87,88]. The Hyeon group has been very active in this research field and they defined necessary characteristics of new contrast agents [89]: positive T_1_ contrast, uptake and distribution, and enhanced circulation times. Key characteristics of nanoparticles such as their small size and diverse chemical modifications facilitate these goals. They succeeded in synthesizing MnO nanoparticles that were water dispersible and biocompatible. The latter feature was achieved by adding a PEG-phospho-lipid shell [90]. These nanoparticles were tested on eight different normal and cancer cells and no toxicity was observed either below ~80 μM or 800 μM, depending on the cell line [89]. Moreover, excellent image quality was reported that was sufficient for neuroscience as well as the management of brain diseases. Finally, the addition of the tumor specific marker Herceptin allowed cancer cell targeting. A higher MnO dose (785 mg Mn mL^−1^) was found to be toxic [91]. Nevertheless, Mn^2+^ ions involved in biological processes as a cofactor do not cause the kidney damage as reported for Gd^3+^ induced NSF [92].

Hollow MnO nanoparticles coated with human serum albumin (HMnONs) may be applied as both MRI contrast enhancing agents and drug carriers [83]. When coated with mesoporous silica (HMnO@mSiO_2_), they exhibit uptake into stem cells and allow imaging in vivo and in vitro for extended periods of time [93].

A higher toxicity of MnO nanoparticles over Fe_3_O_4_ was reported by Gilad et al., and Choi et al. [91,94]. Additionally, pathological changes in liver, lungs and kidneys caused by manganese containing particles have been recorded [95]. Iron oxide nanoparticles exhibit artifacts due to magnetic susceptibility [83] but ultra-small paramagnetic iron oxide nanoparticles [96] also termed as extremely small-sized iron oxide NPs [97] have shown promise as excellent T_1_ MRI contrast agents. The protein corona formed on the NP surfaces [95] has been shown to neutralize charges and make them more biocompatible. Moreover, these particles were cleared by macrophages in the liver and the spleen which supports their medical potential [98].

Increasing research efforts are currently directed at combining diagnostics and therapy, i.e., creating theranostic nanoparticles that may be used in MRI with computer tomography, photodynamic therapy, and drug delivery ([99] and references therein).

## 4. Gadolinium in the Human Organism

### 4.1. Exposure and Uptake

Over geological times, rare earth elements, including Gd, have been released into the environment by erosion and weathering. Any amounts of these elements measured above the naturally-occurring levels are due to anthropogenic activities (see Section 3.2).

Not surprisingly, the highest concentrations of Gd were reported for mining and processing sites. The exposure to the element was especially high at tailing ponds, and transportation and recycling facilities. Furthermore, the workers were subjected to dust and aerosol exposure at the workplace. For example, in the mining region of Baotou, the exposure was recorded to be as high as 56,500 µg g^−1^ with an average level of 4670 µg g^−1^. These values are substantially higher compared to the ones reported for unaffected regions (181 µg g^−1^) [100]. Another report investigating a mining site in Southeastern China correlated increased levels in soil with an uptake into plants. Eventually, the elevated concentrations of 425–1275 µg g^−1^ and 0.06–1.89 µg g^−1^ of rare earth elements in human blood and hair, respectively, were documented and linked to the food supply [101]. A comprehensive overview of the global hot spots may be found in Adeel et al. [102].

For the uptake of Gd^3+^ complexes, conflicting reports were published. In one study, no uptake into families of water plants called macrophytes was found, which was interpreted as low risk for enrichment in the food supply [103]. On the other hand, the uptake of Gd^3+^ complexes into plants grown on contaminated soils or exposed to contaminated water was demonstrated [104]. It has to be stated that uptake also varied between growth stages and plant species. When considering Gd^3+^ uptake into plants, it is important to remember that free Gd^3+^ ion has high affinity to soil and sediments [105], while chelated compounds end up in water ways (see Section 3.2). The impact of Gd^3+^ ions is likely due to competition with Ca^2+^ ions, which may manifest itself in plant development, including cell wall formation, root growth, flowering, or photosynthesis [105]. In terms of food safety, low risk has been reported by Adeel et al., for seafood, vegetables, wheat, and maize [102].

Thus, various pathways may lead to Gd^3+^ uptake into the human body. Interestingly, very poor absorption of Gd^3+^ through the gastrointestinal tract of vertebrates limits accumulation through the food chain. The possible reasons include the formation of insoluble precipitates, binding of Gd^3+^ to food undergoing digestion, and the apparent inability of Gd^3+^ to make use of the Ca^2+^ transporting mechanisms of the intestine (Evans chapter 7) [12]. Much greater quantities of Gd^3+^ find its way into the body through parenteral injection and inhalation. A study by Kramsch et al., reported that ingested La^3+^ exhibited poor absorption following oral administration, however, it did affect arterial Ca^2+^ distributions. Competition of La^3+^ with Ca^2+^ for binding sites at the membrane leads to a reduction of Ca transport across membranes and smaller amounts of Ca bound to macromolecules of the intercellular matrix (e.g., such as the key protein elastin, and the heavily glycosylated proteoglycans) [106].

Despite all the variety of Gd sources discussed above, the major exposure route for humans remains intravenous administering of Gd^3+^-based contrast-enhancing agents for MRI imaging.

### 4.2. Biodistribution and Excretion

Intravenously-administered GBCAs are classified based on whether they distribute in extracellular fluid, intravascular fluid, liver, or blood. There is generally no intracellular distribution of approved GBCAs apart from those that are useful in liver imaging [107].

The dosage of clinically approved GBCAs ranges from 0.1 mmol/kg to 0.3 mmol/kg which translates to about 1 g of Gd^3+^ per person per injection [108]. GBCAs are selected for their ability to increase signal contrast, be stable in vivo, and display complete excretion from the body without prolonged retention [109]. In a study by McLachlan et al., Gadoteridol (Prohance), one of the clinically approved linear GBCAs, was assessed for its pharmacokinetic properties [110]. The study found that intravenous administration of the compound is followed by a rapid transition from the vascular compartments to the extracellular fluid space. Next, it is excreted through glomerular filtration in the kidneys with 94% efficiency within 24 h [110]. Gadobenate dimeglumine (Multihance) and gadoxetic acid disodium (Primovist) belong to a class of GBCAs designed for uptake into the hepatobiliary system. Their uptake into hepatocytes allows liver imaging [108].

Recent evidence of Gd^3+^ deposition in tissues following enhanced MRI scans raised significant concerns about the stability of GBCAs in vivo. The exact mechanism of Gd deposition is not fully understood, however, de-chelation most likely plays a role [15]. Frenzel et al., evaluated the de-chelation rate of GBCAs after a 15-day incubation period at 37 °C in human serum in vitro [111]. The highest rate of de-chelation (20–21%) was found for nonionic linear GBCAs followed by ionic linear GBCAs (1.1–1.9%). Macrocyclic GBCAs were shown to be the most stable with the de-chelation rate not exceeding 0.1%. Another factor that could potentially contribute to the retention of Gd in vivo is transmetallation. Some metal ions present in the body, including Na^+^, K^+^, Mg^2+^, Ca^2+^, Fe^3+^,and Zn^2+^, may compete with Gd^3+^ for the central atom position in the complex. Zn^2+^ was proposed to be the most likely ion that could cause transmetallation of GBCAs as it has a relatively high concentration in the blood and a high affinity for organic ligands [112].

A clinical study found a significant dose-dependent relationship between intravenous GBCA administration and subsequent neuronal tissue deposition [113]. All patients exposed to multiple doses of a GBCA had elevated levels of elemental Gd ranging from 0.1 to 58.8 μg Gd per gram of brain tissue. In particular, one study found an increase in T_1_ signal intensity of the dentate nucleus after multiple GBCA enhanced MRI scans [114]. From the sampled neuroanatomic locations, the dentate nucleus contained the highest median concentrations of elemental Gd. This finding suggests that substantial de-chelation of the GBCA gadodiamide is occurring in patients resulting in Gd^3+^ deposition in the brain. Although no phenotype was connected to these levels of Gd retained in brain tissue, the findings highlight the need for more research into GBCA stability in vivo.

A similar result was observed in the case of bone tissue. Gibby et al., used inductively-coupled plasma atomic emission spectroscopy to quantify Gd^3+^ deposition in bones of patients who have received either Omniscan or Prohance three and eight days before examination [115]. Omniscan, a linear Gd^3+^ containing complex, was found to cause 2.5 times more Gd depositions than the macrocyclic complex Prohance. Further results from Darrah et al., confirmed that Gd incorporates in bones and is retained for over eight years [116]. Advanced analysis by inductively-coupled plasma mass spectrometry (ICP-MS) of bone tissue from patients who have received doses of GBCAs showed Gd^3+^ concentrations of 9.36 nmol/g compared to control patients with 0.03 nmol/g. Contrary to the result from Gibby et al., no differences were observed in bone Gd concentration between patients who received Prohance and Omniscan. These results indicate that GBCAs do not completely clear the body, however it does not reveal the mechanism of retention. The exact chemical species of Gd retained in bone is not understood and more work is required.

The selected examples illustrate that Gd-chelates are not as stable as anticipated and the diagnostic use can lead to deposition in bones and various organs. Implications for human health are discussed in the next section.

### 4.3. GBCA Toxicity at the Organ Level

A well-documented chronic effect of Gd^3+^ is NSF in patients with already reduced renal function. This condition was identified in patients with severe renal impairment whose symptoms included thickening and hardening of the skin of the extremities and an increase in dermal fibroblast-like cells associated with collagen remodeling and mucin deposition [117]. In individuals exposed to Gd^3+^ who exhibit end-stage renal disease, the odds of developing NSF ranges from 20.6% to 41.3% [118]. Although the exact mechanism remains unclear, it has been suggested that the reduced capacity of GBCA excretion due to renal deficiency and the resulting retention increases the likelihood of Gd^3+^ dissociation from its chelating compound over time and, therefore, its toxicity [111]. This hypothesis is supported by the presence of elevated levels of Gd^3+^ in the tissue of patients with NSF who have received contrast-aided MRIs [119,120]. GBCAs that are more stable and, therefore, less likely to dissociate from Gd^3+^ may reduce the odds of NSF occurrence following Gd^3+^ exposure. Thus, the use of macrocyclic and Gd^3+^-chelating compounds with a high thermodynamic stability is recommended for individuals with renal impairment that require contrast aided MRIs. This realization has contributed to a drop in the number of verified cases of NSF in recent times. However, the fact that NSF has been reported to develop anywhere from 24 h to several years after Gd^3+^ exposure remains disconcerting [118]. It is also important to note that not all individuals with end stage renal impairment contracted NSF following Gd^3+^ exposure. Moreover, neither a mechanism, nor proof of causation have been found that directly links exposure of Gd^3+^-containing contrast media and the development of NSF [121].

Considering that the primary route of Gd^3+^ exposure is through intravenously administered GBCAs, Gd^3+^ associated cardiovascular risks have been investigated. In a study by Angeli et al., rat aortic rings were used to assess if Gd^3+^ affects vascular reactivity to contractile responses induced by phenylephrine [122]. Vascular reactivity is modulated in part by extracellular nucleotides such as adenosine triphosphate (ATP). ATP is released from sympathetic nerves and binds to P2X receptors on smooth muscle cells causing vasoconstriction. In contrast, when released from endothelial cells, ATP binds to P2Y receptors and causes vasodilation. This activity can then be regulated by a subset of enzymes called ectonucleotidases with E-NTPDases being the most important. At a concentration of 3 µM, Gd^3+^ is a potent inhibitor of E-NTPDases [123]. Angeli et al. [122] found that ATP hydrolysis was reduced after Gd^3+^ administration, confirming that Gd^3+^ blocked the ability of E-NTPDases to break down ATP. With higher levels of ATP available to activate P2X receptors, an increase in smooth muscle cell contraction is likely. As this includes smooth muscle within the vasculature, there is potential for cardiovascular complications.

Gd^3+^ complexes have been shown to inhibit the function of angiotensin-converting enzyme (ACE) through a transmetalation event with zinc [124]. ACE is a primary component of the renin-angiotensin system which regulates the volume of fluids in the body. However, this inhibitory effect is not directly related to Gd^3+^ but instead its chelating agent. Excess unbound ligand contained within GBCAs are thought to outcompete ACE for zinc resulting in inactivation of the enzyme. This indirectly relaxes blood vessels leading to lower blood pressure.

The accumulation of Gd^3+^ in the central nervous system (CNS) is influenced by a number of individual factors, including age and degeneration of the BBB, as well as underlying diseases, such as von Hippel-Lindau disease (VHL), and Tuberous Sclerosis Complex (TSC) [125,126]. These hereditary tumour syndromes are a result of genetic mutations which, among many issues, result in predisposition for benign tumors. Consequently, patients must undergo frequent MRI screens of the brain, abdomen, and spine, exposing patients to, on average one Gd^3+^ dose per year [127,128]. Kanda et al. found that Gd^3+^ accumulation occurs in the dentate nucleus and globus pallidus of the CNS in patients who have undergone a minimum of five MRI scans [129]. This was demonstrated with unenhanced T_1_-weighted MRI in 381 patients who had undergone between 0 and 30 MRI screens. Signal intensity of the dentate nucleus and globus pallidus showed a positive correlation with the number of previously administered GBCAs and MRI scans. However, this study was performed with linear GBCAs, and other groups have found that macrocyclic GBCAs do not show the same degree of Gd^3+^ accumulation due to their enhanced stability [130,131,132,133]. In addition to the frequency of MRI screens, the nature of the diseases, such as tumour location, may also play a role. Vergauwen et al., determined the prevalence and rate of accumulation by means of unenhanced T_1_-weighted MRI images of dentate nucleus and globus pallidus [126]. This was done on 28 VHL patients and 24 TSC patients. Similar to the findings by Kanda et al., Gd^3+^ accumulated in higher amounts in almost all patients after five MRIs. However, VHL patients showed significantly more accumulation over TSC patients. This may be due to BBB degradation, as VHL onsets later in life and the VHL patients in this study were older than the TSC patients. Individuals with VHL also develop numerous posterior fossa tumors which results in microvascular leakage at the BBB [126].

Little is known about the long-term effects of Gd^3+^ deposition in the CNS of patients with normal renal function. Studies by McDonald et al. and Welk et al. concluded that Gd^3+^ deposition in the brain was not linked to Parkinson’s or any pathological degeneration of brain tissue [113,134]. A survey was performed by Williams and Grimm with the intent of understanding some of the chronic symptoms brought on by Gd^3+^ accumulation that may warrant further studies to determine pathology [135]. All 17 patients reported chronic pain described as aches, paresthesia, or deep bone pain. Twelve patients identified dermal changes such as skin tightening and skin lesions, as well as discoloration. Muscle twitching and loss of strength was also a major concern, occurring in 15 of the patients. Other symptoms ranged from loss of vision to low body temperature and hair loss. All patients reported their symptoms starting within the first month of an MRI procedure, with 59% reporting that symptoms were present on the same day. Many of these symptoms are not linked to any known diseases and might be discarded as short-term side effects, however, they are described here as chronic problems that must be addressed. Investigation into possible mechanisms resulting from Gd^3+^ retention may lead to proper treatment and prevention.

After the overview of organ level toxicity, the subsequent section focuses on molecular interactions and presents model system data comparing Gd^3+^ effects to other metals ions that we have previously investigated.

## 5. Gd Interactions at the Cellular and Molecular Level

Detrimental effects of Gd^3+^ on human organs can be better understood by investigating effects at the cellular and molecular level. The following section provides an overview of Gd^3+^ interactions with major classes of biomolecules, including nucleic acids, carbohydrates, proteins, lipids and their assembly in biological membranes (Section 5.1, Section 5.2 and Section 5.3). The impact on membrane potential is described in Section 5.4. Finally, the biophysical data on model systems composed of biologically relevant lipids as well as lipid mixtures mimicking brain membranes is presented in Section 5.5. The results show that interaction with Gd^3+^ leads to substantial alterations of the structure and function of biological membranes, which, in turn, may cause negative health impacts.

### 5.1. Gd Interactions with Nucleic Acids and Carbohydrates

Various interactions of Gd^3+^ within the cell must be considered in order to understand its cytotoxicity. The cellular environment contains numerous biomolecules, including nucleic acids, nucleosides, glycoproteins, and glycolipids, to name a few. They all represent potential targets for Gd^3+^ which may subsequently interrupt crucial processes and biological pathways.

As nucleic acids are polyanionic, they may interact with positively charged ions including Gd^3+^. The complexation with metals leads to alteration of DNA secondary structure by inducing bends, kinks, and local helix unwinding, as well as by forming adducts and inter- or intra-strand crosslinks [136]. These structural changes may disrupt proper DNA functionality and interaction with proteins. Cytotoxicity of Gd^3+^ complexes has been reported in HL-60 myeloid cells [137]. To determine potential mechanisms, inorganic salts of Gd(III) were introduced into DNA samples and the structural changes were determined by monitoring the signature peaks in Raman spectra of biomolecules. For instance, the ratio of peaks at 683 and 670 cm^−1^ was measured to be less than one in the presence of Gd^3+^, indicating that the DNA molecules had altered their conformation from the native B to the A form. The A conformation of DNA is characterized by an extra base-pair per turn compared to the B form, and a deeper major groove, which inhibits the interaction with proteins. The helix-turn-helix motif is often found in transcription proteins in order to recognize specific nucleotides in the major groove prior to transcription. When the DNA is in its A form, this vital process is blocked. Gd^3+^ was also reported to cause DNA fragmentation via an unknown mechanism, which induced apoptosis in tumor cells [55]. This can be potentially viewed as beneficial for the use of Gd^3+^-based drugs in therapeutics, however, in the context of unwanted Gd^3+^ exposure, this a dangerous effect.

In the cytoplasm, the main nucleic acid of concern is RNA. It comes in a variety of forms, from the most well-known messenger RNA (mRNA) and transfer RNA (tRNA), which are used for translating genetic information into proteins, to regulatory forms, such as micro RNA, which functions in the regulation of gene expression by silencing or inducing degradation of mRNA. Reports have shown that Gd^3+^ interacts with yeast phenylalanine tRNA (tRNA^Phe^) at loops in the RNA formed by sharp turns [138]. The first binding site was found at residues 8 and 9, where interaction occurs between Gd^3+^ and the backbone phosphate oxygens. In this case, the octahedral geometry that Gd^3+^ prefers is saturated by the addition of 4 water molecules. The second site is located in a loop between residues 20 and 21 and is coordinated by the same interactions. Electron density calculations also revealed that Gd^3+^ can interact with the phosphate on residue 14 of one tRNA^Phe^ and the phosphate on residue 56 of a neighbouring tRNA^Phe^. This study, along with others, reveal that Gd^3+^ does not interact with the bases of nucleic acids directly, but with the phosphate oxygens [139,140], which is not surprising considering the phosphate backbone is negatively charged. Gd^3+^ ability to link adjacent tRNAs is of concern as this can interfere with tRNAs binding to ribosomes and protein synthesis. However, the binding and dissociation constants of Gd^3+^ with tRNA^Phe^ must be examined further to determine the extent of damage this might cause.

**Figure 5:**
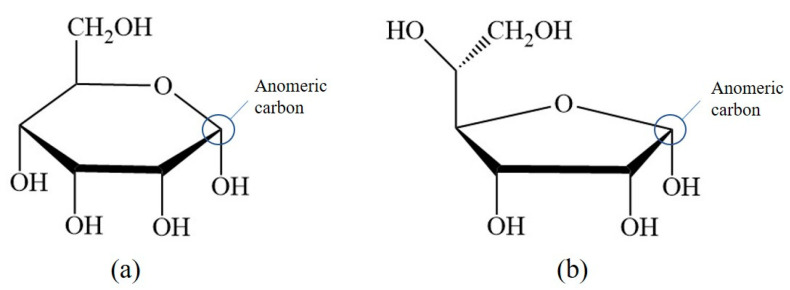
Structures of monosaccharides (**a**) α-pyranose and (**b**) α-D-allofuranose displaying axial positioning on the anomeric carbon.

Lanthanides have relatively low affinity for simple uncharged saccharides and can only bind if three -OH groups on the sugar ring are in a specific conformation [141]. This limits binding to the α-anomer configuration found in monosaccharides such as α-d-allofuranose and α-pyranose [142] (Figure 5). This selectivity is known to alter the anomeric population of saccharides [141], that is, the percentage of saccharides with the -OH group on the anomeric carbon in either the axial or equatorial position (Figure 5). The addition of a carboxyl group significantly increases the binding potential of Gd^3+^, therefore, there is a higher affinity for glycoproteins over saccharides. Chemical shifts from NMR studies on the glycoproteins α-d-glucopyranuronate and α-d-galactopyranuronate confirm bidentate chelation with the ring oxygen and carboxyl oxygen, similar to how Gd^3+^ complexes to chelating agents [143].

Sialic acid is a nine-carbon amino sugar with a carboxyl group positioned at the anomeric carbon. It is commonly found on the surface of cell membranes, playing a role in cell recognition and signaling [144]. Its high abundance and negatively charged carboxyl group make it a potential binding target for trivalent ions such as Gd^3+^. *N*-acetylneuraminic acid (Neu5Ac) is one of the most common sialic acids. It is found at the terminal ends of glycoproteins and glycolipids in the cell membrane. This particular sialic acid is overexpressed in tumours and serves as a biomarker in assays to detect malignancies such as colon cancer and brain tumors [145,146,147]. Consequently, one study has measured the binding location and kinetics of the Gd-chelates (Gd-DO3A) with Neu5Ac for this purpose [148]. It was found that the carboxylate of Neu5Ac coordinates to the Gd^3+^ ion in the complex with a binding constant of 151 ± 4 [148]. It was also reported that saccharides such as d-fructose, d-galactose, and d-mannose also interact with the Gd^3+^ complex. The binding constant with fructose was the highest of these sugars, at 646 ± 14, indicating that strong competition exists in a cellular environment [148]. While Gd-chelate binding can be used for clinical purposes such as MRI, the potential of undesired unspecific binding must also be considered, as Gd-chelates used in traditional MRI scans may bind to, and interfere with, some of these important signaling molecules.

### 5.2. Effects on Proteins (Channels), Channels, Ca^2+^ Mimic

Gd^3+^ has an ionic radius similar to that of Ca^2+^ but a higher valency. As a result, Gd^3+^ competes with Ca^2+^ in vivo, leading to many adverse effects on molecular and cellular processes. These effects stem from Gd^3+^ acting as an antagonist towards calcium-dependent protein channels and enzymes. An example of this is with L-type calcium channels, which are responsible for the excitation-contraction coupling of skeletal, smooth, and cardiac muscle as well as the regulation of neurotransmitters [149].

A vast body of Gd^3+^ cytotoxicological studies have been conducted on Kupffer cells (KC), the major macrophage in liver tissue. The proper functioning of these cells is essential to preventing various pathological conditions, such as total hepatic ischemia and sepsis [150,151]. Palasz et al., has found out that the incubation of rat KC with 1.0 mmol/L GdCl_3_ led to blockage of K-type calcium channels, preventing both passive and receptor-mediated influx of Ca^2+^ [152]. As a result, many calcium-dependent pathways were inhibited, including the synthesis of the prostanoid prostaglandin E_2_, which is critical to produce an inflammatory cascade in response to septic shock.

Gd^3+^ uptake by KCs has also been reported to inhibit cellular transport processes, such as phagocytosis, and alter the expression of cytokines (small signaling peptides) and cytokine-regulated transcription factors [153,154]. A study by Ruttinger et al., reported that injection of 10 mg/kg of GdCl_3_ into rats induced a KC blockade and reduced phagocytic activity [155]. Two mechanisms have been proposed which act congruently to produce this effect. First, GdCl_3_ is soluble at low pH, but may precipitate at physiological pH, forming aggregates which interfere with KC activity [153,156]. This was confirmed by intravital fluorescence microscopy in which the distribution of 1.1 μm fluorescent latex particles was recorded over 5 min. A reduction in phagocytic activity was observed in the presence of GdCl_3_, as the number of particles engulfed by KCs was reduced by ~11%. Second, Gd^3+^ competes with extracellular calcium, inhibiting the first phase of phagocytosis by blocking calcium binding sites on the surface of the membrane [157]. With KC unable to internalize GdCl_3_ precipitates, the KC blockade was maintained. Further experiments were performed to determine hepatocellular function by measuring the levels of cytokines, enzymatic activity, and bile flow 24 h after GdCl_3_ treatment. Two cytokines, IL-6 and TNF-α, were at highly elevated activity levels in blood serum, 4624 ± 1205 U/mL and 74 ± 8 U/mL, respectively, compared to 114 ± 33 U/mL for IL-6 and no detectable TNF-α activity in the controls. Enzymatic activity of alanine aminotransferase and aspartate aminotransferase were increased two- and three-fold, respectively, while bile flow was reduced from 3.0 ± 0.1 μL min^−1^ g^−1^ to 2.4 ± 0.1 μL min^−1^ g^−1^. This alteration in hepatic activity is indicative of liver injury and hepatocellular dysfunction [156]. While the study could not fully determine the mechanism of hepatic alteration, it is believed that phagocytosis of the GdCl_3_ aggregates activates the KCs, leading to release of cytokines and an increase metabolic activity, ultimately leading to morphological and functional alterations of parenchymal cells that perform the organ function. This was explored further by D.A. Badger et al., which found that hepatocytes were also directly affected by GdCl_3_ induced alteration of KCs [158], as increased concentrations of TNF-α and IL-6 levels reduced expression and activity of cytochrome P450 [159]. This family of enzymes is essential for the breakdown of many external molecules in order to protect cells. GdCl_3_ injection at 10 mg/kg led to a 30% decrease in P450 concentration in male rats and a 20% decrease in female rats. They proposed three mechanisms for this decrease. The first one is an increase in the inhibitory cytokines, TNF-α and IL-6. It was also theorized that GdCl_3_ could decrease the production of stimulatory cytokines, such as interleukin-4, or enter the hepatocyte directly and interfere with P450 in an unknown method. Regardless, it is clear that Gd^3+^ directly effects KCs which results in adverse effects at the organ level.

Gd^3+^ cytotoxicity in neurons is another area of interest, as they contain many ion channels and calcium-dependent receptors that are known Gd^3+^ targets. Gd^3+^ deposition in the brain has also been reported and its accumulation may lead to debilitating outcomes [160,161]. A study by Huettner et al. examined the antagonistic effects of Gd^3+^ on the GluR6 kainate receptor in dorsal root ganglion cells and hippocampal cells [162]. These receptors respond to the important neurotransmitter glutamate, while the term kainate refers to an antagonist isolated from algae. This type of receptor acts as a mediator for presynaptic and post synaptic responses and malfunction has been associated with disorders such as ischemia (inadequate blood supply) [163,164,165]. Gd^3+^ was added at concentrations of 1.6 μM, 6.3 μM, and 25 μM followed by 200 μM of kainate to measure whole-cell currents at −80 mV and +50 mV. Complete current blockade was achieved at 25 μM for both membrane potentials with an IC_50_ of 2.3 μM at −80 mV and 3.5 μM at +50 mV. In order to determine if inhibition is caused by Gd^3+^ binding within the conducting pore of the channel, the membrane potential was increased under the assumption that the antagonistic effect would decrease, as both the transmembrane field and outward flow of cations would aid in removal of the Gd^3+^ blocker. In this experiment, recovery kinetics were recorded by applying 10 μM of Gd^3+^ to the receptor with 1 mM kainate under the two membrane potentials. Results indicated that the potency of the block was slightly weaker at +50 mV, but not enough to conclude that Gd^3+^ enters the channel. An increase in kainate concentration did not affect the degree of antagonism, therefore, competitive inhibition was also ruled out, leaving the possibility that binding occurs at an external site which results in some allosteric reduction of affinity at positive membrane potentials. These examples show that Gd^3+^ is a potent inhibitor of GluR6 receptors while other studies have shown that it is even more potent towards voltage gated calcium channels with an IC_50_ of 163 nM [166].

Gd^3+^ deposition in the brain is extremely toxic as it can block influx of Ca^2+^ into the presynaptic membrane. A potential action reaching the pre-synapse will open voltage-gated calcium channels, causing a rapid influx due to the calcium concentration gradient. This triggers the release of neurotransmitters, which travel through the synaptic cleft to the post-synapse, carrying on the signal. The amount of calcium influx is proportional to the amount of neurotransmitter released, so blockage of calcium channels can weaken or terminate the signal prematurely. In order to study the inhibitory effects of Gd^3+^ on Ca^2+^ influx, a team performed a Monte Carlo cell simulation study on a model synapse surrounded by Ca^2+^ and Gd^3+^ at varying ratios [167]. The presence of Gd^3+^ had a significant effect on the diffusion of Ca^2+^. At a Ca^2+^:Gd^3+^ ratio of 6:1, all calcium channels are blocked within 0.200 s. At a 1:1 ratio, complete blockage occurs in just 0.019 s. Both ions compete at first to reach the channels, however once Gd^3+^ binds, it completely restricts entry of Ca^2+^. This result is also reflected by the amount of calcium ions in the pre-synapse and the amount of neurotransmitter released. At a 6:1 ratio, the maximum amount of Ca^2+^ ions that diffused in was 1592, approximately 300 ions less than in the absence of Gd^3+^. At a 1:1 ratio the amount drops to only 622 Ca^2+^ ions. The corresponding amount of neurotransmitter released is 2098 without Gd^3+^, and 1868 and 590 at ratios of 6:1 and 1:1, respectively. The results of this study are incredibly relevant for MRI procedures, as potential Gd^3+^ leakage can block calcium-channels and disrupt vital signals.

Another crucial class of channels that Gd^3+^ can block are stretch-activated (SA) ion channels. These channels respond to mechanical stress from the cell membrane, altering their conformation between open and closed states to regulate the flow of ions [168]. Gd^3+^ has been reported to block gravity sensing in plant roots, which relies on SA channels [169]. To determine the means by which Gd^3+^ blockage occurs, Yang and Sachs examined the effects on SA channels in *Xenopus* oocytes [170]. They found that Gd^3+^ has three different concentration-dependent effects on SA channels. The first is a reduction in channel open time upon addition of 0–5 μM Gd^3+^. The reciprocal of the open time is a linear function of the Gd^3+^ concentration, which corresponds to the traditional closed-open-block kinetic model [171]. The blocking rate was determined from this function to be 1.6 × 10^8^ M^−1^ and remained independent of voltage, indicating that blockage occurs outside the membrane electric field. Channel current was also reduced in the presence of Gd^3+^ across the entire voltage range of −30 mV to −200 mV. Compared to the normal current-voltage curve, 5 μM of Gd^3+^ shifts the curve 26 mV to the left without altering the slope conductance. This suggests that the drop in conductance is a result of surface charge neutralization by Gd^3+^. The trivalent nature of Gd^3+^ makes it effective at screening negative surface charge and subsequently decreasing the local concentration of permeant monovalent cations [172]. At a Gd^3+^ concentration of 10 μM, channel activity is completely inhibited. This is not caused by blocking the open channel, as extrapolation of the linear plot to 10 μM results in an open time of 0.53 ms. Similarly, the channel current does not reach zero upon saturation. Therefore, Gd^3+^ induces a third type of blocked state with a lifetime longer than the 300 s duration of the experiment. The steep concentration dependence from 5 to 10 μM that results in complete blockage suggest a Hill coefficient greater than 7, and the authors of this paper theorize that a cooperative transition occurs in the channel when Gd^3+^ occupies a certain amount of negatively charged sites. Comparison of Gd^3+^ inhibition with other SA channels revealed that Gd^3+^ is not a generic channel blocker. For example, K^+^-selective SA channels in rat astrocytes and T lymphocytes are not blocked by 10 μM of Gd^3+^, but SA channels in yeast and *Xenopus* myocytes are [170,173]. Despite this, it is still a potent blocker of mechanoreceptive transducers.

Among the many receptors Gd^3+^ may interfere with, the G protein-coupled receptor family, including the metabotropic glutamate receptor 1α (mGluR1α), is of great interest as it plays a role in synaptic plasticity and initiates various cell responses. One such response is an increase in intracellular Ca^2+^ and the important second messenger cyclic adenosine monophosphate (cAMP), via coupling to G proteins, which operate as cellular switches [174,175]. This G protein-coupled receptor is unique amongst the other classes as it has a homodimer conformation, a large extracellular domain, and is sensitive to polyvalent cations like Ca^2+^ and Gd^3+^ [176]. This receptor acts as an on/off switch, resting in an “open” state. When glutamate binds to one of the lobes a conformational change is induced in which it enters a closed-open active state. However, in the presence of Gd^3+^, glutamate may also bind to the other lobe and the receptor enters a second active state in which both lobes are ‘closed’. These alternate active states result in different intracellular domain (ICD) conformations, where the G-protein interacts, thereby changing the intended signal response. A fluorescence approach, Foerster resonance energy transfer analysis, was used to deduce the dimeric rearrangement of the ICD. Results showed that low concentrations of Gd^3+^ (10–300 μM) cause rearrangement of the ICD into the second active state, while high concentrations (>300 μM) induce an inactive state. In its native state, mGluR1α interacts with the G protein subunits, Gq and Gs, activating both pathways resulting in increased [Ca^2+^] and [cAMP], respectively. However, the induced second active state by Gd^3+^, alters the mGluR1α ICD such that it only prefers Gq. Therefore, only intracellular [Ca^2+^] was increased. This type of effect can be damaging to the cell, as extracellular signals which would induce the Gs pathway, may now only receive the Gp response.

Extensive literature has covered the inhibitory effects of Gd^3+^ on calcium channels and pumps by binding to the calcium-binding sites of these proteins. This is also true for transient receptor potential channels (TRPV6), which are inhibited by Gd^3+^ at concentrations above the micromolar range. A study by Kovacs et al., found that the expression of TRPV6 at the plasma membrane increased the transport of rare earth elements including Gd^3+^ [177].

### 5.3. Introduction to Lipids and Model Membranes

In contrast to other macromolecules, metal interactions are investigated with lipid ensemble rather than individual lipid molecules since lipids readily self-assembly into double layers with the hydrophobic tails hidden from the surrounding aqueous environment and the hydrophilic heads facing the extracellular and intracellular compartments. Membranes serve many roles functioning as permeability barriers, separators of cellular compartments, and sites of signaling and regulated transport. Moreover, vesicular transport is important within cells as well as in key processes such as synaptic signal transmission [178].

**Figure 6:**
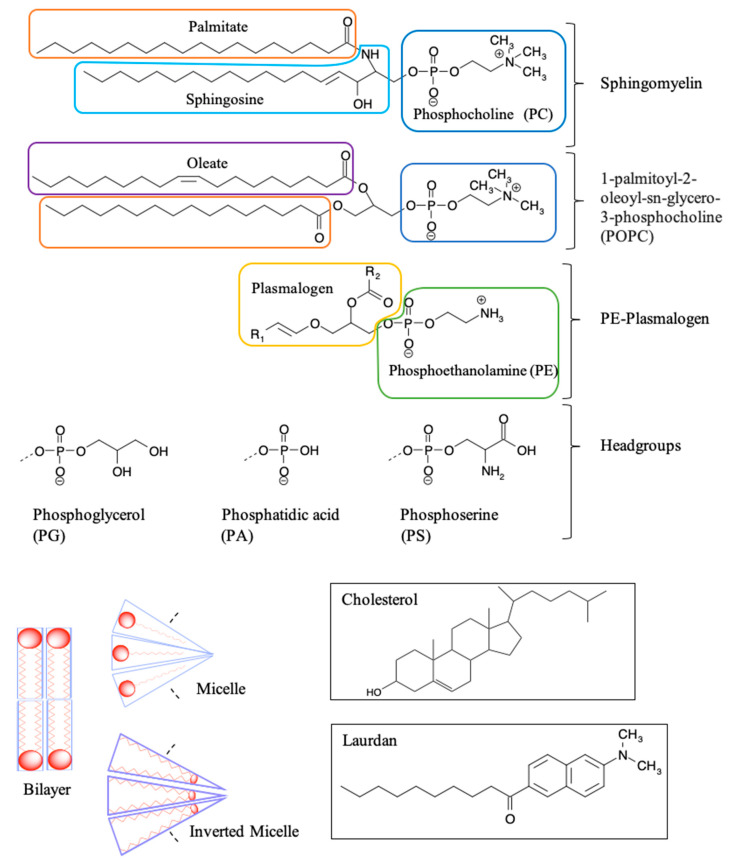
Structures of acyl chains and head groups commonly found in lipid membranes, as well as the structures of cholesterol and Laurdan. (R_1_, R_2_ = chain lengths on plasmalogen vary). Packing arrangement of phospholipids, based on acyl-chain/head group composition. Cylinder-shaped phospholipids form bilayers and wedge-shaped phospholipids form micelles/inverted micelles.

Lipids are amphipathic molecules with well-defined hydrophilic and hydrophobic parts. Biological membranes are composed of varying amounts of phospholipids, glycolipids and sterols [179]. The main mammalian lipid classes possess either a glycerol or sphingosine backbone (see Figure 6). The most important representatives of the former class are phosphatidylcholines (PC), while the most abundant member of the latter is sphingomyelin (SM) (see Figure 6). Each lipid has two hydrophobic chains that become building blocks for the hydrophobic core between the two polar leaflet surfaces. Ring-based sterols, like cholesterol (see Figure 6), are an important factor in modulating membrane characteristics such as rigidity/fluidity and the lateral organization within a leaflet [180]. Membranes are fluid and flexible structures under physiological conditions and adopt the so-called liquid-crystalline phase. This terminology reflects the fact that, although the overall structure remains fluid, well defined lipid and/or protein domains may be found within two leaflets. This description is an improvement on the original fluid mosaic membrane model by Singer and Nicolson [181] that treats a fluid sea of lipids as a matrix to embed proteins. More recent models also highlight the importance of rigid lateral islands, termed lipid rafts, as platforms where certain proteins could be segregated in order to interact with ligands and participate in signaling cascades [182].

Another key characteristic of mammalian membranes is the asymmetric distribution of lipids within the outer and the inner leaflet of the bilayer [183]. The outer leaflet is enriched in PCs and SM, the major building blocks of membranes while SM also adds rigidity due to longer hydrophobic acyl chains and the ability to form hydrogen bonds in the interface region. Both lipid classes are zwitterionic with the negative charge on the phosphate linker neutralized by the positive charge on the nitrogen of the choline group. Mammalian membranes also contain negatively-charged lipids, such as phosphatidylserine (PS), which are predominantly localized at the inner leaflet of the bilayer [183]. PS has two negative charges, one on the phosphate at the interface and the other on the carboxyl in the headgroup. It also contains a positive charge on the amine within the headgroup, resulting in an overall net charge of −1. PS is known to be involved in signaling [184] and has been shown to form clusters in the presence of cations such as Ca^2+^ [185].

In terms of their 3-D structure, membranes form bilayers that become more organized and rigid at lower temperatures adopting the so-called gel phase. The transition between the gel and liquid-crystalline phase is a reversible process that occurs at the characteristic phase transition temperature (Tm). The latter phase constitutes membranes under physiological conditions [186]. The Tm of a lipid depends on the length and saturation of the acyl chains. Lipids with saturated chains have higher Tm due to stronger van der Waals interactions, whereas lipids with unsaturated chains require more space due to the presence of double bonds in the cis conformation. As a result, the increased distance between lipid molecules leads to an overall reduced lipid packing, and, consequently, diminished attraction and lower Tm [187].

Moreover, lipids can adopt so-called non-bilayer structures that are important for cellular processes such as membrane fusion. The propensity of lipids to adopt these structures is well-described by the shape model [186]. If the headgroup is comparable in size to the acyl chain, the overall shape resembles a cylinder (see Figure 6). These lipids can be best arranged in double layers and sheets, i.e., the bilayer structure. If the headgroup regions are smaller than the acyl chains, inverted micelles are formed (see Figure 6). Reversing the geometry and making the headgroup region larger compared to the acyl chain, will lead to self-assembly of these lipids into micelles (see Figure 6). The ability of lipids to form micelles have been identified as a critical process in membrane fusion.

These parameters, membrane fluidity, Tm, lipid packing, and phase changes are tools to assess the impact of metals on model and biological membranes (e.g., Sule et al. [188]).

One possible source of Gd^3+^ induced cytotoxicity is its complexation with lipids in membranes. Lanthanides, in general, and Gd^3+^ in particular, are known to have high affinity for phospholipid bilayers [189]. Binding of positively-charged ions neutralizes negatively-charged lipids and affect the membrane potentials (see Section 5.4). Ion binding often reduces membrane fluidity [188,190] and may also affect the phase transition temperature. Lipid model systems are used to assess these interactions. They are termed liposomes, which is primarily used in this review, but are also referred to as vesicles, as reflected in the abbreviations for model systems discussed in the next paragraph.

Liposomes with one bilayer can be made smaller than 100 nm and are termed small unilamellar vesicles. Large unilamellar vesicles (LUVs) typically range in size between 100 and 200 nm. LUVs are used for fluorescence ion binding and membrane fluidity measurements via dye release, whereas liposomes with several bilayers, termed multilamellar vesicles (MLVs), are more suitable for thermodynamic characterization of Tm, regarding the range of the transition, and the energy involved. These parameters may be elucidated using differential scanning calorimetry (DSC).

#### Membrane Interactions of Gadolinium

For example, DSC data showed that the addition of Gd^3+^ to DPPC MLVs with a Gd^3+^/lipid molar ratio of 0.5 stabilized the packing of a fully saturated DPPC (two 16 carbon side chains) in the gel phase and increased the phase transition temperature by 5 °C [191]. These findings were further supported by freeze-fracture electron microscopy. The trivalent ions La^3+^ and Gd^3+^ were found to replace Ca^2+^ from DPPC liposomes, which was also shown by DSC [191]. Furthermore, Gd^3+^ increased the order of phospholipids and induced a structural change in the orientation of the headgroup relative to the surface, from mostly parallel, in the case of the control, to mostly perpendicular in the presence of Gd^3+^ [192]. Additionally, a continuous increase in the Tm was observed as Gd^3+^ was added at concentrations ranging from 1 mM to 1 M. Finally, aggregation of DPPC vesicles was detected by dynamic light scattering (DLS) at a metal concentration above 200 mM [192]. A study by Tanaka et al. investigated the effects of Gd^3+^ on the shape of Giant unilamellar vesicles composed of DOPC [193]. They found out that Gd^3^+ at concentrations as low as 10 µM induced a shape change from discoid to a bucket like (stomatocyte) structure in 7 s. For comparison, it took 12 s for La^3+^ at a concentration of 100 μM to induce a comparable change [193].

In addition to the zwitterionic PC membranes, similar studies were performed to investigate Gd^3+^ interactions with anionic lipid systems. Averbakh et al., exposed suspensions of multilamellar dimyristoylphosphatidylserine (DMPS) liposomes to low concentrations of GdCl_3_ [194]. The addition of metal ions led to partial aggregation of the liposomes and the authors proposed metal interaction with accessible PS in the outer leaflet was the main cause of aggregation.

In addition to model membranes, complex cells were investigated to study permeability of molecules, ions, and small particles, considering that the control of transmembrane transport is essential for proper cellular function. Gianulis et al., investigated the inhibitory mechanism of Gd^3+^ on electropermeabilization [195]. They subjected Chinese hamster ovary cells to nanosecond electric pulses that would create pores to allow the uptake of the otherwise membrane impermeant dyes YO-PRO-1 and propidium. The addition of Gd^3+^ prevented the dye uptake and caused the electropores to rapidly reseal or shrink even after the ion was removed. The binding of the ions to the polar lipid groups results in dehydration and makes the membranes more hydrophobic, which may further facilitate pore closure [195].

Cheng et al., investigated the impact of Gd on membrane permeability by exposing human erythrocytes to various Gd species: Gd^3+^, [Gd(Citrate)_2_]^3−^, and Gd^3+^+EDTA [196]. They used atomic force microscopy to image the membrane topography upon addition of Gd^3+^. Low concentrations of the ion (1.0–10 µM) induced the formation of lipid domains within the membrane surface whereas higher concentrations (25 µM) resulted in pore formation and enhanced permeability. Gd(III) citrate induced domain formation only, as no pores were observed even at a concentration as high as 25 µM. This suggests that pore formation is correlated to the presence of free Gd^3+^. This hypothesis was further supported by imaging the membrane after washing it with EDTA. As a chelator, EDTA reduced the concentration of Gd^3+^, which, in turn, led to resealing of pores.

Finally, Lopatina et al., showed that addition of Gd^3+^ resulted in calcium-independent neurotransmitter release by inducing vesicle-based exocytosis [197]. Synaptosomes isolated from rat cerebral hemispheres were loaded with the dye acridine orange and suspended in a medium containing 100–300 µM Gd^3+^. Gd^3+^ induced a rapid increase of fluorescence intensity due to the release of dye in the absence of calcium.

The binding of charged metal ions also neutralizes charges on the membrane, which, in turn, affects membrane potentials as discussed in the next section.

### 5.4. Gd^3+^ Effect on Membrane Potential

Gd^3+^ belongs to a group of ions that have high affinity to lipid membranes. The interaction of these ions with hydrophilic headgroups of phospholipids, in addition to a partial neutralization of the surface charge, may have a significant impact on the membrane, including changes in lipid packing, orientation, and phase transitions. It is still debated whether Gd^3+^ blocks ion channels of different nature as a result of the ion binding to specific proteins or due to incorporation of Gd^3+^ into the lipid bilayer, which, in turn, results in major structural changes in the membrane in the vicinity of the ionic channels.

A well-established method to investigate interaction of different ions with lipid membranes is through obtaining the membrane surface zeta potential. This can be done by measuring electrophoretic mobility of liposomes using commercially available instruments. The electrophoretic mobility μ is used to calculate the zeta potential ζ at a so-called slipping plane of the electrolyte double layer using the Smoluchowski equation:

(4) $$\mu =\frac{\zeta \varepsilon \epsilon_{0}}{\eta }$$

where *ε* is the dielectric constant, $\epsilon_{0}$ is the permittivity of free space, and η is the dynamic viscosity.

The concentration of ions at some arbitrary distance x from the charged surface follows the Boltzmann distribution [198]:

(5) $$c_{i}\left(x\right)=c_{i}\left(\infty \right)\exp\left(-\frac{ez_{i}\phi \left(x\right)}{kT}\right)$$

while the electrical potential ϕ(x) at this distance is a function of charge density and may be obtained by solving the Poisson equation:

(6) $$\rho \left(x\right)=-\varepsilon \epsilon_{0}\left(\frac{d^{2}\phi \left(x\right)}{dx^{2}}\right)=\underset{i}{\sum }\frac{z_{i}e}{N_{A}}c_{i}\left(x\right)$$

Combining the Boltzmann and Poisson equations, one may obtain an expression for the distribution of the electrical potential near a charged surface, which reveals that the potential decays exponentially with a characteristic length *k*:

(7) $$\tanh\left(\frac{ez\phi \left(x\right)}{4kT}\right)=\exp\left(-\kappa x\right)\tan h\left(\frac{ez\phi \left(0\right)}{4kT}\right)$$

where $k=\sqrt{2e^{2}c/\varepsilon \epsilon_{0}kT}$ is the inverse Debye length. Equation (7) only works for monovalent electrolytes. For electrolytes containing ions of higher valences, the valence z of the dominant ion should be used and the ion concentration needs to be replaced with the ionic strength of the solution.

Additionally, the surface charge density may be obtained from Equations (5) and (6):

(8) $$\sigma =\sqrt{8kT\varepsilon \epsilon_{0}c}\;\sin h\left(\frac{ez\phi \left(x\right)}{2kT}\right)$$

However, if more than one kind of ion is present in the electrolyte, the Graham equation is used to calculate the surface charge:

(9) $$\sigma^{2}=2kT\varepsilon \epsilon_{0}\underset{i}{\sum }c_{i}\left(\exp\left(-\frac{ez_{i}\phi \left(0\right)}{kT}\right)-1\right)$$

Finally, since the surface charge density σ is due to adsorbed ions, the Gouy–Chapman–Stern model can be combined with the Langmuir isotherm in order to obtain binding constants *K*_1_ and *K*_2_ [199]:

(10) $$\frac{\sigma }{S}=\frac{\left(z_{2}-1\right)K_{2}c_{2}\left(0\right)-1}{1+K_{1}c_{1}\left(0\right)+K_{2}c_{2}\left(0\right)}$$

Equation (10) takes into account competitive adsorption of two different cations, one with the valence of 1 and the other with the valence of $z_{2}$, to anionic phospholipid headgroups. The concentrations $c_{1}\left(0\right)$ and $c_{2}\left(0\right)$ represent the ion concentrations at the surface. When phospholipids are zwitterionic and the membrane is neutral overall, the contribution of monovalent cations may be neglected and Equation (10) is further reduced to:

(11) $$\frac{\sigma }{S}=\frac{z_{2}K_{2}c_{2}\left(0\right)}{1+K_{2}c_{2}\left(0\right)}$$

It is important to note that in applying the Langmuir isotherms, a 1-to-1 stoichiometry between ions and phospholipids is assumed. The maximum density of the binding sites *S* can be estimated from the surface area that one lipid headgroup occupies on the surface of the membrane.

Although this approach has proven to be useful in studying the interaction between ions and membranes, it only provides information on changes in the diffuse part of the double layer surrounding the membrane. In order to gain deeper understanding of how the interaction affects the lipid organization, it would be instructive to measure the dipole component of the membrane potential, i.e., the component associated with the molecular structure, organization, and orientation of lipids constituting the membrane. In other words, one would need to be able to measure changes in the electrical potential in the part of the membrane that is not accessible by counterions. Such a method called intramembranous field compensation was developed and used to investigate the interaction of Gd^3+^ with PC and PS lipid membranes [200,201]. It has been shown that the interaction of Gd^3+^ with the negatively-charged PS lipid membranes induce a substantial change in the membrane dipole potential that emerges in a non-linear fashion with the concentration of the ion. In particular, a significant jump was observed when 30% of the binding sites became occupied. Such observations would be consistent with the formation of domains with a different degree of lipid packing, which inevitably alters thermodynamical and mechanical properties of the membrane.

### 5.5. Introduction to Metal Membrane Interactions

When discussing the interaction of metal ions with model membrane systems, one key aspect that needs to be taken into consideration is the speciation of metals under the selected experimental conditions, i.e., the presence of metal ion complexes with counterions This concept may be illustrated by comparing the two divalent ions Cd^2+^ and Hg^2+^. Depending on the pH and the amount of sodium chloride added, the concentrations of different species that co-exist in solution at equilibrium vary [202]. For example, at pH 7.4 and in the presence of 100 mM NaCl, Cd in solution is split between 45% of CdCl^+^, 35% of CdCl_2_ and 20% of Cd^2+^, whereas under the same conditions Hg exists as 35% of HgCl_2_, 30% of HgCl_3_^−^ and 35% of HgCl_4_^2−^ [203]. The latter observation is particularly interesting: not only do chloride anions completely neutralize the positive charge on Hg, but they also turn it into an overall negatively-charged species under these experimental conditions. Speciation can be calculated by using the software Visual MINTEQ based on chemical equilibrium data [204].

The effect of speciation and a strong role of electrostatics on the interaction of metal ions with lipids have been demonstrated by the Prenner group when preferential interaction of Cd and Hg with negatively and positively charged membranes was investigated [205]. Naturally, both Cd and Hg exist as divalent cations in aqueous solution. However, in the presence of moderate concentrations of sodium chloride, Hg cation forms complexes with chloride anions. The resulting negatively-charged complexes were shown to have strong binding affinity to positively-charged membranes. Furthermore, upon binding, the metals induced membrane rigidity and in some cases membrane fusion and liposome aggregation [205].

Similar experiments are currently being conducted with Gd^3+^ in the Prenner lab, with the rationale that the metal under physiological pH and salt conditions speciates into Gd^3+^ at 86.8%, and GdOH^2+^, GdCl^2+^, and GdNO_3_^2+^ at 8.6%, 3.5%, and 1.1%, respectively (speciation data calculated using Visual MINTEQ [204]).

In addition to the overall lipid membrane charge, the chemical structure of individual lipids has been shown to be an important factor. In particular, Cd exhibited stronger affinity to the negatively charged PS compared to PA membranes that are also negatively charged (see Figure 6 for lipid structures). Furthermore, both liposome systems were precipitated by Cd in contrast to liposomes made of negatively-charged phosphatidylglycerol (see Figure 6). The authors suggested that the neutral glycerol headgroups sterically shield the charged phosphates, thus, preventing their interaction with metal ions, and, consequently, liposome fusion [205].

Exposing liposomes to individual metals versus metal mixtures also had different effects. For example, aggregation of liposomes induced by Cd may be partially reversed by adding Hg to the mixture [206].

These insights laid the foundation for further studies of metal interaction with lipids in complex biological extracts and red blood cell membranes, confirming Cd interaction with negatively charged lipids and showing that Hg targets and cleaves the enolether bond in plasmalogens (Figure 6) [203].

Another important experimental factor to consider is potential unspecific binding of metal ions to common organic buffers, and, as a consequence, a loss of metal from solution by precipitation. For example, Cd-phosphate has a very low solubility. The impact of such experimental conditions, such as buffer selection, on metal membrane interactions was reviewed by Payliss et al. [190].

#### Membrane Interactions of Gadolinium

The previous discussion was intended to establish the framework for the on-going investigation of the effects of Gd^3+^ interaction with lipids that are being conducted in the Prenner group. Some of the preliminary and unpublished results are presented below.

One way of probing the lipid organization within the membrane is by monitoring the fluorescence of laurdan (Figure 6) as a function of temperature [205]. Briefly, the long hydrophobic chain of the fluorophores ensures its insertion into the hydrophobic core of the membranes, while the polar and solvent sensitive naphthalene group is located at the interphase. The emission peak of the dye depends on the extent of hydration in this part of the bilayer. Tighter lipid packing results in fewer water molecules surrounding the fluorophore and an emission peak at 440 nm. As the membrane becomes more fluid and more water molecules are able to penetrate into the interphase space of the membrane, the emission peak shifts bathochromically and the maximum emission is observed at 490 nm. The ratio of fluorescent intensities as shown in the formula was termed generalized polarization (*GP*) [207]:

(12) $$GP=\frac{I_{440}-I_{490}}{I_{440}+I_{490}}$$

Higher values of *GP* indicate more rigid membranes. Thus, any changes in *GP* compared to metal free controls may serve as an indicator to lipid packing and organization as a result of interaction with metal ions.

**Figure 7:**
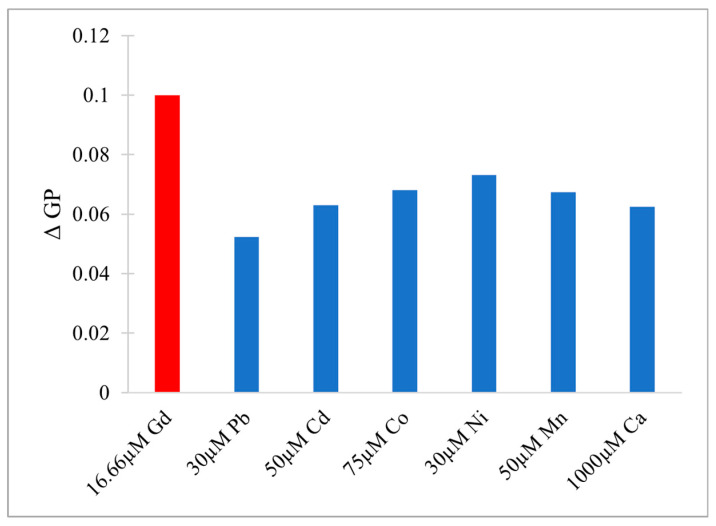
Generalized polarization of POPS liposomes in the presence of various heavy metals. The graph is compiled from different studies that were designed to characterize single metals and thus the concentration ranges used vary.

The data in Figure 7 show the changes in *GP* (ΔGP) observed when 1-palmitoyl-2-oleoyl phosphatidylserine (POPS) liposomes are exposed to Gd^3+^ ions. Furthermore, we compare this data to the results obtained in similar experiments in the Prenner group but with different metal ions. The comparison includes Ca, a known competitor with Gd^3+^ for binding sites, as well as Cd, which is recognized as one of the most toxic elements in nature.

The US Agency for Toxic Substances and Disease (ATSD) maintains a list of toxic elements or compounds that are used on a larger scale [208]. We have selected the following positively-charged ions or metal chloro-complexes that have been recognized in the 2019 list: Pb (#2); Cd(#7); Co (#52), Ni (#58); (Mn#140), in order to evaluate their impact on lipid membranes. Except for a minor rigidifying effect of Pb on POPC membranes, all metals showed no impact on the zwitterionic, but overall neutral POPC liposomes. In contrast, all exhibited interactions and binding with the negatively charged POPS, which is known as a very important lipid in cell signaling [184] (Figure 7).

The observed changes in GP infer an enhanced membrane rigidity upon interaction with various metal ions. Gd^3+^ shows the highest change at the lowest overall concentration compared to other metals. At this point, it seems particularly important to emphasize that an increased membrane rigidity potentially implies structural lipid reorganization, formation of new domains, and changes in the membrane electrostatic properties, fluidity, and elasticity. All of the above may seriously affect proper functioning of the cell. Thus, interaction of Gd^3+^ with lipids in cell membranes may cause much more serious implications than currently understood.

The data is compiled from several studies using different concentration ranges. All metals at higher concentrations than Gd^3+^ still induce smaller changes in GP. Metal concentration increases are approximated to reflect how many fold higher amounts were used, while the reduced GP increases is presented in percent (rounded values).

Order of increasing metal concentrations:

Gd (1×) < Pb, Ni (2×) < Cd, Mn (3×) < Co (4.5) << Ca (60×)

Corresponding order of reduced GP increases:

Gd (100%) > Pb (60%) > Ni (30%) < Cd (40%)~Mn (40%)~Co (35%)~Ca (40%)

All values, but especially the much higher amount of Ca, still induced lower GP changes, strongly illustrating the potency of Gd^3+^ to impact POPS membranes.

Since Gd^3+^ is used as a contrast enhancing agent for MRI in the brain, several preliminary experiments have been conducted with SM liposomes and brain polar lipid extracts. Brain membranes are known to be enriched in SM lipids [209], which, on average, have longer side chains compared to PC and have the ability to form hydrogen bonds in the interphase. These bonds are formed between the hydroxyl group at the sphingosine base and the amide linkage on the side chain. Consequently, SM rich membranes are more rigid than PC membranes and SMs have been shown to form lipid domains in mixtures with POPC [210]. Moreover, they are considered building blocks of lipid rafts, islands of high rigidity where certain proteins are sequestered in order to facilitate their interactions. A lipid raft is depicted as a yellow island in the graphical abstract of this review. These membrane structures serve a variety of cellular functions, such as signaling and adhesion, and have been used by pathogens to enter cells [211].

**Figure 8:**
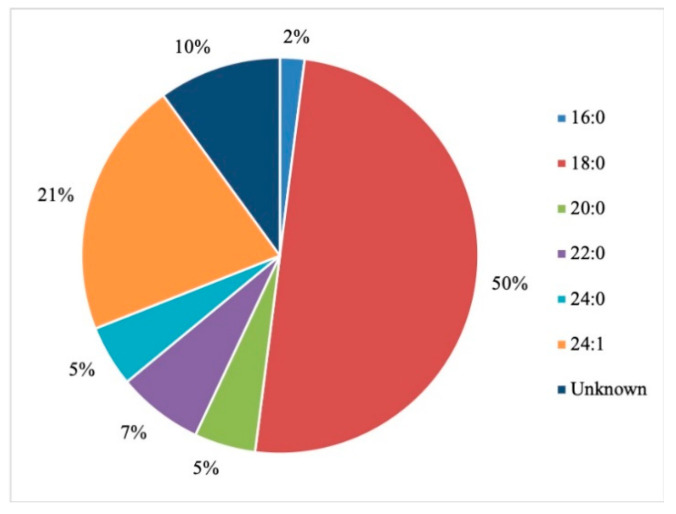
Average fatty acid distribution of porcine brain SM. Adapted from Avanti polar lipids; https://avantilipids.com/product/860062.

The composition of commercially available porcine brain SM is given in Figure 8. Most of the side chains are saturated fatty acids, whereby 50% are stearic acid (18 carbons length) and only 21% are monounsaturated 24:1 (Figure 8).

**Figure 9:**
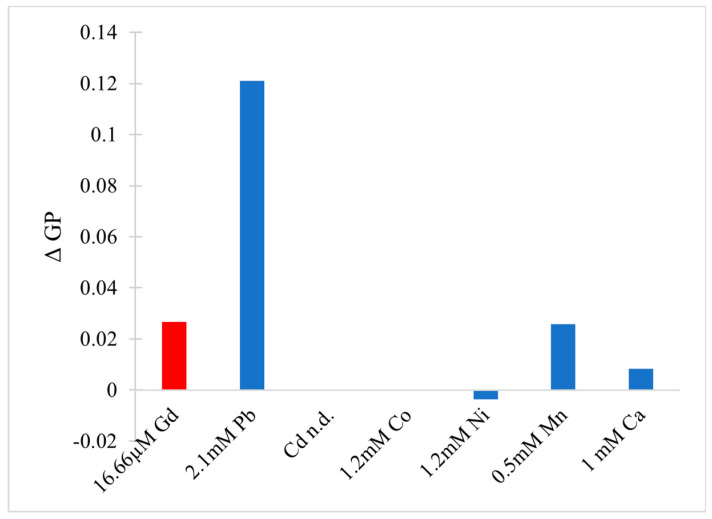
Generalized polarization of brain SM liposomes in the presence of various heavy metals.

This composition results in rigid membranes with high GP values of 0.30 versus 0.02 for a more fluid POPS membranes discussed above. Based on our previous work, metals, in general, do not induce significant changes in an already rigid membrane (Figure 9).

Only Gd, Pb, and Mn show appreciable effects. The Pb-assisted rigidification peaks at a metal concentration that is ~125 times higher compared to the concentration of Gd^3+^ required to cause the same magnitude of effect. The changes due to addition of Mn are comparable to the ones observed with Gd^3+^ but require a 30× higher metal concentration (Figure 9). Ca at ~60× higher concentration only induced a minor increase of GP (~1/3 of the Gd^3+^ increase) (Figure 9).

**Figure 10:**
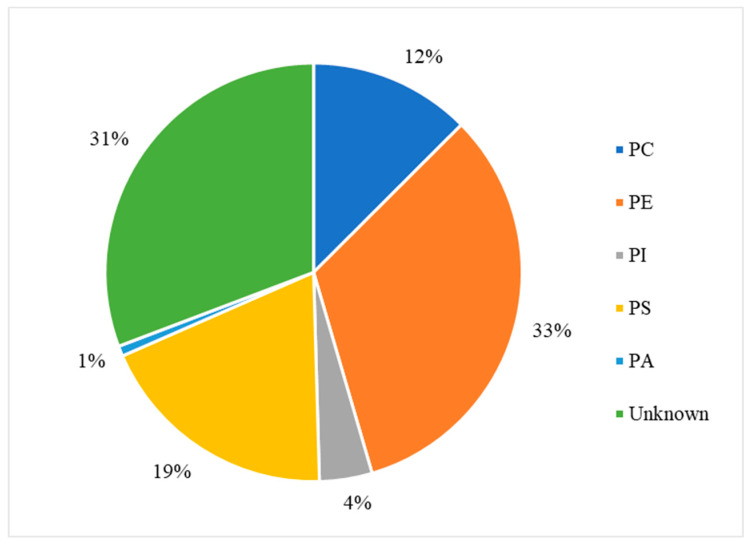
Lipid composition of porcine brain polar extract. Adapted from Avanti polar lipids; https://avantilipids.com/product/141101.

The next system investigated was a more complex matrix, porcine brain polar lipid extract, whose composition is shown in Figure 10.

This extract is made from total brain extract and contains ~20% negatively-charged lipids as potential metal binding targets. The total extract is produced with chloroform/methanol whereas the polar extract is isolated after treatment with acetone and diethylether according to the product information provided by the supplier Avanti Polar Lipids.

**Figure 11:**
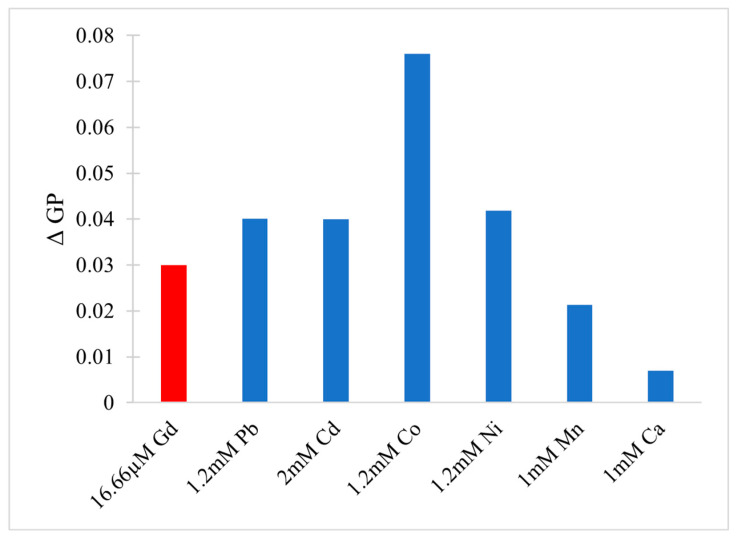
Generalized polarization of BPE liposomes in the presence of various heavy metals.

Although Gd^3+^ was used at a concentration substantially lower compared to other metal ions, it still induced the largest change in GP (Figure 11). Metal concentrations increases are approximated to reflect how many fold higher amounts were used, while the reduced GP increases is presented in percent (rounded values).

Order of increasing metal concentrations:

Gd (1×) << Mn, Ca (60×) < Pb, Co, Ni (75×)

Corresponding order of GP changes:

Co (250%) > Pb, Cd, Ni (130%) > Gd (100%) > Mn (70%) > Ca (30%)

Considering substantial concentration differences, Gd^3+^ had the relative strongest impact.

In conclusion, Gd^3+^ had the strongest effect in all 3 different model systems by inducing more membrane rigidity at lower concentrations compared to other metals as well as Ca^2+^.

The more complex systems also exhibit packing problems between the various lipids. We have shown for Cd and Hg that the extent of their interactions with biomimetic red blood cell (RBC) lipid mixtures of PC/PC-PE/PC-PS/PC-PE-PS-cholesterol changes with the composition of the lipid matrices. Furthermore, the addition of metal mixtures showed different effects than single metal experiments. This aspect is not addressed often but suggests much more complex scenarios to be considered in future work [212].

Subsequently, we assessed the ability of the various metal ions to induce liposome aggregation as determined by dynamic light scattering.

The same Gd concentration as in the GP experiments was used. No change in size was observed, although, concentrations slightly above 25 μM resulted in complete precipitation of the POPS liposomes.

An increase in metal concentrations is approximated to reflect the amounts used, while the size increase (nm) indicates metal effects compared to controls.

Order of increasing metal concentrations:

Gd (1×) < Pb (2×) << Co, Ni (38×) <Mn, Ca (60×) << Cd (125×)

Corresponding order of liposome size changes:

Cd (200 nm) > Ni (150 nm) > Ca (120 nm) > Co, Mn (100 nm) >> Pb (20 nm)

**Figure 12:**
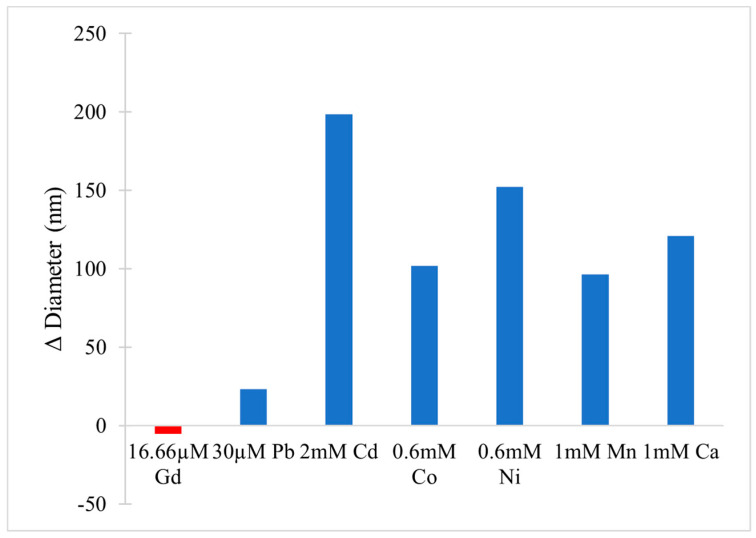
Change in size of POPS liposomes in the presence of various heavy metals. Measured by DLS.

Size increases of more than 100 nm indicate liposome aggregation (Cd, Ni, C, Co, Mn) and we have previously reported Cd induced aggregation and precipitation [205]. The moderate increase induced by Pb in the liposome diameter suggests liposome swelling but not aggregation (Figure 12).

**Figure 13:**
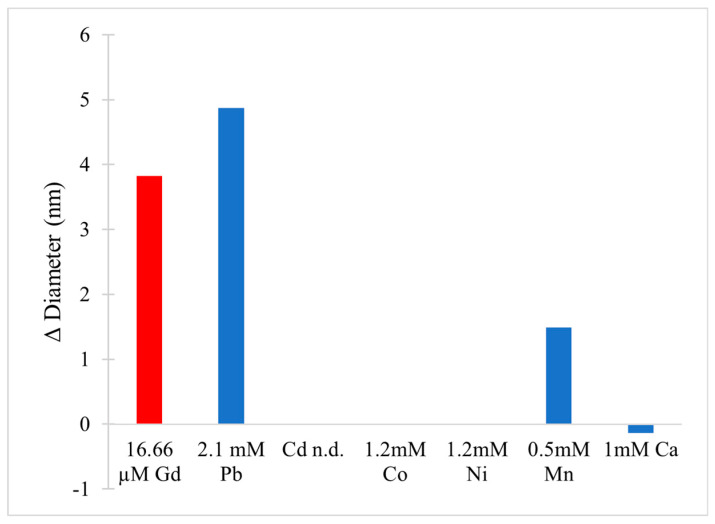
Change in size of brain SM liposomes in the presence of various heavy metals. Measured by DLS.

Size changes in brain SM liposomes are shown in Figure 13. The size of the control liposomes was 116 nm. The low concentration of Gd^3+^ resulted in a minor change of ~4 nm. About 130× higher Pb concentration resulted in a comparable increase of 5 nm whereas a 30× higher amount of Mn had a very moderate effect of 1.5 nm. Cd was not determined, whereas Co, Ni, and Ca did not induce statistically relevant changes.

**Figure 14:**
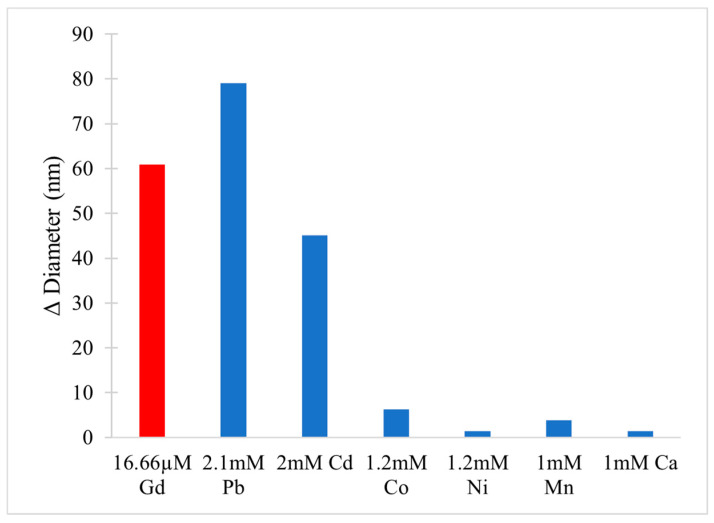
Change in size of Brain Polar Extract liposomes in the presence of various heavy metals. Measured by DLS.

Finally, size changes in the more complex brain polar extract were compared (Figure 14).

A diameter increase from 116 to 176 nm was induced by a low Gd^3+^ concentration. The increase is not sufficient to be explained by aggregation of liposomes but potentially results from liposome fusion.

Metal concentrations increases are approximated to reflect how much metal was used, while the size increase (nm) indicates metal effects compared to controls.

Order of increasing metal concentrations:

Gd (1×) << Mn, Ca (60×) < Co, Ni (70×) << Cd (120×) < Pb (130×)

Corresponding order of liposome size changes:

Pb (80 nm) > Gd (60 nm) > Cd (45 nm) >> Co (7 nm)~Mn (4 nm)

The observed changes for Pb, Cd and Gd are not enough to account for aggregation. The minor changes for Co and Mn suggest very moderate swelling, whereas Ni and Ca had no effect

We view this preliminary data for model membranes composed of biologically relevant lipids and complex lipid mixtures as a call for a thorough and detailed investigation of cellular processes upon exposure of cells to Gd^3+^ ions. We find this call timely and urgent considering that Gd^3+^ and GBCAs are not even included by ATSD on the list of dangerous elements that comprises a total of 275 entries, yet Gd^3+^ shows to affect the lipid membrane structure to a substantially greater extent compared to widely accepted and recognized culprits.

Moreover, metal effects have been demonstrated in complex biological membranes as Cd and Pb induced rigidity in RBC at 1–10 mM concentrations in contrast to Ca [213]. Native RBC membranes contain mainly PC and SM in the outer leaflet, whereas PE, PS or PI are found on the inner leaflet. Inside out RBC membranes exhibited increased fluidity and the formation of echinocytes, characterized by spike like protrusions suggesting metal insertion in the outer leaflet [214]. Metal effects are not limited to lipids but also include interactions with proteins, and complex effects like apoptosis have been reported for Cd in kidney cells [215]. Trivalent ions like Al have been implicated in toxicity for many organs [216] and as a main facilitator of iron induced lipid peroxidation [217]. This is achieved by increased lipid packing as demonstrated by GP analysis [217]. Tighter lipid packing supports the propagation of the chain reaction [218]. These authors also showed for a series of metals (Sc, Ga, In, Y, La, Be) that their capacity to induce fusion in PC/PS (60/40) liposomes was directly correlated with their ability to support Fe induced lipid peroxidation [218]. Both Al and Pb have been implicated with many toxic effects, for a comprehensive review see Verstraten et al., (2008) [219]. Pb is able to induce phase separation in PC/PS liposomes and also promoted peroxidation. This ability was linked to its capacity to facilitate liposome aggregation and fusion [220]. Finally, vanadium-containing complexes that induced membrane fluidity were able to induce aggregation of luteinizing hormone receptors, resulting in a signaling response that is otherwise initiated by binding of the hormone ligand [221], representing a significant impact on signaling. This novel model of metal induced signaling was also reviewed [222]. Membrane effects of these vanadium complexes were stronger than the effects of the V(IV) solution, which is interesting as membrane studies of Gd^3+^ focused on the metal ion itself [221]. Another interesting fact is the report that vanadium(V) has structural similarity to the phosphate group and can act as an inhibitor for the phosphorylases [223]. Finally, a variety of metal complexes of Pt, Cr, Ru, Rh, V, and Mo have been identified recently for use in medical applications as they interact with a variety of biologically-relevant molecules.

The previous paragraph briefly illustrated the broad range and complexity of metal membrane interactions and their impact in biological systems. Nevertheless, the factors addressed in our model membrane work, namely the binding to negatively-charged membranes, the modulation of membrane fluidity, and the ability to induce aggregation and fusion have been shown to be relevant for the other metals discussed above and contribute to their impact on membrane structure and function that are key for human health and well-being.

The trivalent nature of Gd^3+^ and its complex coordination chemistry allow many of these interactions and the preliminary data indicates a strong potential of this metal to significantly impact biological membranes, but much more work will be necessary as was emphasized above.

## 6. Summary and Concluding Remarks

Gadolinium is a peculiar example of an element that living organisms would rarely encounter naturally if it was not for anthropogenic activity. It is the human drive for technological advances that turned the metal found in trace amounts in ores into a front-man in many industries and an element whose concentrations substantially exceed natural levels in industrialized regions of the world.

Numerous highly technological Gd-based applications have been launched in the energy sector and information technologies, and unique chemical properties of the element allowed Gd to find its niche in diagnostics and therapeutics. Although the advantages of using Gd in all these fields are indisputable, the benefits start being overshadowed by an increasing body of evidence pointing at potentially negative impacts that Gd exerts on human health and the environment. Some of the examples discussed in this review are related to the use of Gd^3+^ in MRI, in particular, the metal accumulation in human organs and elevated levels detected in the environment due to excretion. As a result, the general population in some regions becomes exposed to Gd at concentrations much higher than the natural background level. It is particularly disturbing considering that this second-hand exposure through the consumption of water and food involves unchelated Gd^3+^ which is more toxic. Some known effects of Gd on important biological processes include its ability to mimic Ca^2+^ and interfere with many physiological roles, including signaling, which have not been thoroughly explored and certainly require attention of the scientific community. For example, the data presented in this review on how the membrane organization changes when lipids interact with Gd^3+^ unambiguously demonstrates a significant degree of induced alterations, which, in turn, may have detrimental consequences.

Overall, long-term effects of exposing the human body to Gd^3+^, and particularly the effects of metal accumulation, is poorly understood. Unfortunately, it is not a unique observation that technological advances often happen on a much faster timescale compared to our ability to grasp in full their health and environmental impacts. This review does not attempt to provide a comprehensive description of research activities and progress in terms of our understanding of the problem—we acknowledge that many meaningful contributions in the vast amount of literature have not been included. Neither does it aim at drawing an unequivocal conclusion about the use of Gd^3+^ in terms of weighing the indisputable medical advances against the unquestionable negative impact on the environment and potentially human health.

Instead, the review invites interested readers to consider the issue from different angles and continue the discovery of multifaceted chemical and biochemical characteristics of Gd in numerous applications. It is also intended to outline remediation efforts and encourage a quest for the development of alternatives. Most importantly, however, the review aims at unveiling and highlighting the effects of Gd^3+^ in the areas that have been poorly investigated, namely the interaction of the metal with lipids and cell membranes. The available synopsis of biophysical experimental data, although still scarce, suggests that Gd^3+^ can produce effects comparable in magnitude to the ones caused by most toxic metals, except Gd^3+^ can give rise to these effects at much lower concentrations. These findings should raise concerns and result in enhanced efforts aimed at better understanding Gd^3+^ behaviour in the human body to enhance health and well-being.

## Abbreviations

ACE, angiotensin-converting enzyme; ATP, adenosine triphosphate; ATSD, US agency for Toxic Substances and Disease; BBB, blood-brain-barrier; RBC, red blood cell; cAMP, cyclic adenosine monophosphate; CNS, central nervous system; DLS, dynamic light scattering; DMPS, dimyristoylphosphatidylserine; DOPC, 1,2-dioleoyl-sn-glycero-3-phosphocholine; DOTA, dodecanetetraacetic acid; DPTA, diethylenetriaminepentaacetic acid; DSC, differential scanning calorimetry; GBCA, gadolinium based contrast agent; Gd, gadolinium; GP, generalized polarization; ICD, intracellular domain; KC(s) Kupffer cell(s); LUVs, large unilamellar vesicles; MGd, Motexafin gadolinium; mGluR1a, metabotropic glutamate receptor 1a; MLV, multilamellar liposome; MRI, Magnetic Resonance Imaging; MSN, Mesoporous silica nanoparticles; Neu5Ac, *N*-acetylneuraminic acid; NSF, nephrogenic systemic fibrosis; PAAS, Post-Archean Australian Shale; Tm, phase transition temperature PC, phosphatidylcholine; PEG, polyethylene glycol; POPS, 1-palmitoyl-2-oleoyl phosphatidylserine; PS, phosphatidylserine; ROS, reactive oxygen species; SA, stretch-activated; SM, sphingomyelin; tRNA^Phe^, yeast phenylalanine transfer RNA; TSC, Tuberous Sclerosis Complex; VHL, von Hippel-Lindau disease.

## References

[1] Barbalace K. Periodic Table of Elements: Gadolinium—Gd. EnvironmentalChemistry.com. https://environmentalchemistry.com/yogi/periodic/Gd.html.

[2] Rogowska J., Olkowska E., Ratajczyk W., Wolska L. (2018). Gadolinium as a new emerging contaminant of aquatic environments. Environ. Toxicol. Chem..

[3] U.S. Geological Survey (2019). Mineral Commodity Summaries.

[4] Evans C.H. (1990). Biochemistry of the Lanthanides.

[5] Bünzli J.-C.G. (2006). Benefiting from the Unique Properties of Lanthanide Ions. Accounts Chem. Res..

[6] Laing M. (2009). Gadolinium: Central Metal of the Lanthanoids. J. Chem. Educ..

[7] Sherry A.D., Caravan P., Lenkinski R.E. (2009). Primer on gadolinium chemistry. J. Magn. Reson. Imaging.

[8] Darnall D.W., Birnbaum E.R. (1973). Lanthanide ions activate α-amylase. Biochemistry.

[9] Evans C.H. (1990). Interactions of Lanthanides with Tissues, Cells, and Cellular Organelles. Biochemistry of the Lanthanides.

[10] Daumann L.J. (2019). Essential and Ubiquitous: The Emergence of Lanthanide Metallobiochemistry. Angew. Chem. Int. Ed..

[11] Rinard P., Reilly R.D., Ensslin N., Smith H. (1991). Neutron Interactions with Matter. Passive Nondestructive Assay of Nuclear Materials.

[12] Evans C.H. (1990). The Occurrence and Metabolism of Lanthanides. Biochemistry of the Lanthanides.

[13] Sugimae A. (1980). Atmospheric concentrations and sources of rare earth elements in the Osaka area, Japan. Atmos. Environ..

[14] Bau M., Dulski P. (1996). Anthropogenic origin of positive gadolinium anomalies in river waters. Earth Planet. Sci. Lett..

[15] Rogosnitzky M., Branch S. (2016). Gadolinium-based contrast agent toxicity: A review of known and proposed mechanisms. BioMetals.

[16] Ebrahimi P., Barbieri M. (2019). Gadolinium as an Emerging Microcontaminant in Water Resources: Threats and Opportunities. Geosciences.

[17] Ringbom A. (1963). Complexation in Analytical Chemistry.

[18] Ringbom A., Kolthoff I.M., Elving P.J. (1963). Treatise in Analytical Chemistry.

[19] Weinmann H.J., Brasch R.C., Press W.R., Wesbey G.E. (1984). Characteristics of gadolinium-DTPA complex: A potential NMR contrast agent. Am. J. Roentgenol..

[20] Port M., Idée J.-M., Medina C., Robic C., Sabatou M., Corot C. (2008). Efficiency, thermodynamic and kinetic stability of marketed gadolinium chelates and their possible clinical consequences: A critical review. BioMetals.

[21] Adding L.C., Bannenberg G.L., Gustafsson L.E. (2001). Basic Experimental Studies and Clinical Aspects of Gadolinium Salts and Chelates. Cardiovasc. Drug Rev..

[22] Caravan P., Lauffer R.B., Edelman R., Hesselink J., Zlatkin M., Crues J. (2005). Contrast Agents: Basic Principles. Clinical Magnetic Resonance Imaging.

[23] Mann J.S. (1993). Stability of Gadolinium Complexes In Vitro and In Vivo. J. Comput. Assist. Tomogr..

[24] White D.H., DeLearie L.A., Moore D.A., Wallace R.A., Dunn T.J., Cacheris W.P., Imura H., Choppin G.R. (1991). The Thermodynamics of Complexation of Lanthanide (III) DTPA-Bisamide Complexes and Their Implication for Stability and Solution Structure Investigative Radiology. Investig. Radiol..

[25] Meyer D., Schaefer M., Bonnemain B. (1988). Gd-DOTA, A Potential MRI Contrast Agent Current Status of Physicochemical Knowledge. Investig. Radiol..

[26] Idée J.M., Port M., Robic C., Medina C., Sabatou M., Corot C. (2009). Role of thermodynamic and kinetic parameters in gadolinium chelate stability. J. Magn. Reson. Imaging.

[27] Brücher E., Werner K. (2002). Kinetic Stabilities of Gadolinium(III) Chelates Used as MRI Contrast Agents. Contrast Agents I Magnetic Resonance Imaging.

[28] Dumazert J., Coulon R., LeComte Q., Bertrand G.H.V., Hamel M. (2018). Gadolinium for neutron detection in current nuclear instrumentation research: A review. Nucl. Instruments Methods Phys. Res. Sect. A Accel. Spectrom. Detect. Assoc. Equip..

[29] Yang M., Liu X., Zhang Z., Song Y., Bai L. (2019). Effect of Adding Rare Earth Elements Er and Gd on the Corrosion Residual Strength of Magnesium Alloy. Open Phys..

[30] Lamastra F.R., Bianco A., Leonardi F., Montesperelli G., Nanni F., Gusmano G. (2008). High density Gd-substituted yttrium iron garnets by coprecipitation. Mater. Chem. Phys..

[31] Kuanr B.K. (1997). Effect of rare-earth Gd3+ on instability threshold of YIG. J. Magn. Magn. Mater..

[32] Bass M. (1995). Handbook of Optics, Volume I—Fundamentals, Techniques, and Design.

[33] Rim K.T., Koo K.H., Park J.S. (2013). Toxicological Evaluations of Rare Earths and Their Health Impacts to Workers: A Literature Review. Saf. Health Work.

[34] Caravan P., Ellison J.J., McMurry T.J., Lauffer R.B. (1999). Gadolinium(III) Chelates as MRI Contrast Agents: Structure, Dynamics, and Applications. Chem. Rev..

[35] Dong X., Tahir M.A., Zhang L., Schäfer C.G., Xu N. (2019). Gadolinium-containing polymer microspheres: A dual-functional theranostic agent for magnetic resonance imaging and cancer therapy. New J. Chem..

[36] Šimečková P., Hubatka F., Kotouček J., Knötigová P.T., Mašek J., Slavík J., Kováč O., Neča J., Kulich P., Hrebík D. (2020). Gadolinium labelled nanoliposomes as the platform for MRI theranostics: In vitro safety study in liver cells and macrophages. Sci. Rep..

[37] Gao T., Xu L., Kuang Y., Xiong D., Gao T. (2017). Gadolinium-based nanoscale MRI contrast agents for tumor imaging. J. Mater. Chem. B.

[38] Mishra R., Su W., Pohmann R., Pfeuffer J., Sauer M.G., Ugurbil K., Engelmann J. (2009). Cell-Penetrating Peptides and Peptide Nucleic Acid-Coupled MRI Contrast Agents: Evaluation of Cellular Delivery and Target Binding. Bioconjug. Chem..

[39] Heckl S., Pipkorn R., Waldeck W., Spring H., Jenne J., Von Der Lieth C.-W., Corban-Wilhelm H., Debus J., Braun K. (2003). Intracellular visualization of prostate cancer using magnetic resonance imaging. Cancer Res..

[40] Nguyen T.D.T., Marasini R., Rayamajhi S., Aparicio C., Biller D., Aryal S. (2020). Erythrocyte membrane concealed paramagnetic polymeric nanoparticle for contrast-enhanced magnetic resonance imaging. Nanoscale.

[41] Peters T., Grunewald C., Blaickner M., Ziegner M., Schütz C.L., Iffland D., Hampel G., Nawroth T., Langguth P. (2015). Cellular uptake and in vitro antitumor efficacy of composite liposomes for neutron capture therapy. Radiat. Oncol..

[42] Sato T., Hashizume M., Hotta Y., Okahata Y. (1998). Morphology and proliferation of B16 melanoma cells in the presence of lanthanoid and Al3+ ions. BioMetals.

[43] Liu H., Yang X., Wang K. (2006). Effects of Lanthanide Ions (La3+, Gd3+ and Yb3+) on Growth of Human Normal Liver Line 7701 and Human Cervical Carcinoma and the Effect of Cell Apoptosis Induced by Lanthanide. J. Chin. Rare Earth Soc..

[44] Fu L.-J., Li J.-X., Yang X.-G., Wang K. (2009). Gadolinium-promoted cell cycle progression with enhanced S-phase entry via activation of both ERK and PI3K signaling pathways in NIH 3T3 cells. J. Biol. Inorg. Chem..

[45] Zhang Y., Fu L.-J., Li J.-X., Yang X.-G., Yang X.D., Wang K. (2009). Gadolinium promoted proliferation and enhanced survival in human cervical carcinoma cells. BioMetals.

[46] Long X.-H., Yang P.-Y., Liu Q., Yao J., Wang Y., He G.-H., Hong G.-Y., Ni J.Z. (2011). Metabolomic profiles delineate potential roles for gadolinium chloride in the proliferation or inhibition of Hela cells. BioMetals.

[47] Ushikubo S., Aoyama T., Kamijo T., Wanders R.J.A., Rinaldo P., Vockley J., Hashimoto T. (1996). Molecular Characterization of Mitochondrial Trifunctional Protein Deficiency: Formation of the Enzyme Complex Is Important for Stabilization of Both α- and β-Subunits. Am. J. Hum. Genet..

[48] Gardocki M.E., Jani N., Lopes J.M. (2005). Phosphatidylinositol biosynthesis: Biochemistry and regulation. Biochim. Biophys. Acta Mol. Cell Biol. Lipids.

[49] Skwarek L.C., Boulianne G.L. (2009). Great Expectations for PIP: Phosphoinositides as Regulators of Signaling During Development and Disease. Dev. Cell.

[50] Ding S.-J., Li Y., Tan Y.-X., Jiang M.-R., Tian B., Liu Y.-K., Shao X.-X., Ye S.-L., Wu J., Zeng R. (2004). From Proteomic Analysis to Clinical Significance: Overexpression of Cytokeratin 19 Correlates with Hepatocellular Carcinoma Metastasis. Mol. Cell. Proteom..

[51] Dos Santos M.A., Borges J.B.R., De Almeida D.C.G., Curi R. (2004). Metabolism of the microregions of human breast cancer. Cancer Lett..

[52] Schafer F.Q., Buettner G.R. (2001). Redox environment of the cell as viewed through the redox state of the glutathione disulfide/glutathione couple. Free Radic. Biol. Med..

[53] Vertuani S., Angusti A., Manfredini S. (2004). The Antioxidants and Pro-Antioxidants Network: An Overview. Curr. Pharm. Des..

[54] Hashemy S.I., Ungerstedt J.S., Avval F.Z., Holmgren A. (2006). Motexafin Gadolinium, a Tumor-selective Drug Targeting Thioredoxin Reductase and Ribonucleotide Reductase. J. Biol. Chem..

[55] Ye L., Shi Z., Liu H., Yang X., Wang K. (2011). Gadolinium induced apoptosis of human embryo liver L02 cell line by ROS-mediated AIF pathway. J. Rare Earths.

[56] Beneke R., Geisen C., Zevnik B., Bauch T., Müller W.-U., Küpper J.-H., Möröy T. (2000). DNA Excision Repair and DNA Damage-Induced Apoptosis Are Linked to Poly(ADP-Ribosyl)ation but Have Different Requirements for P53. Mol. Cell. Biol..

[57] Candé C., Cecconi F., Dessen P., Kroemer G. (2002). Apoptosis-inducing factor (AIF): Key to the conserved caspase-independent pathways of cell death?. J. Cell Sci..

[58] Martin R.F., Feinendegen L.E. (2016). The quest to exploit the Auger effect in cancer radiotherapy—A reflective review. Int. J. Radiat. Biol..

[59] Yokoya A., Ito T. (2017). Photon-induced Auger effect in biological systems: A review. Int. J. Radiat. Biol..

[60] Matsumoto K., Saitoh H., Doan T.L.H., Shiro A., Nakai K., Komatsu A., Tsujimoto M., Yasuda R., Kawachi T., Tajima T. (2019). Destruction of tumor mass by gadolinium-loaded nanoparticles irradiated with monochromatic X-rays: Implications for the Auger therapy. Sci. Rep..

[61] Brünjes R., Hofmann T. (2020). Anthropogenic gadolinium in freshwater and drinking water systems. Water Res..

[62] Kulaksız S., Bau M. (2011). Anthropogenic gadolinium as a microcontaminant in tap water used as drinking water in urban areas and megacities. Appl. Geochem..

[63] Zhu Y., Hoshino M., Yamada H., Itoh A., Haraguchi H. (2004). Gadolinium Anomaly in the Distributions of Rare Earth Elements Observed for Coastal Seawater and River Waters around Nagoya City. Bull. Chem. Soc. Jpn..

[64] McLennan S.M. (1989). Chapter 7. Rare Earth Elements in Sedimentary Rocks: Influence of Provenance and Sedimentary Processes. Geochemistry and Mineralogy of Rare Earth Elements.

[65] De Boer J.L.M., Verweij W., Van Der Velde-Koerts T., Mennes W. (1996). Levels of rare earth elements in Dutch drinking water and its sources. Determination by inductively coupled plasma mass spectrometry and toxicological implications. A pilot study. Water Res..

[66] Kümmerer K., Helmers E. (2000). Hospital Effluents as a Source of Gadolinium in the Aquatic Environment. Environ. Sci. Technol..

[67] Roth F., Lessa G.C., Wild C., De Kikuchi R.K.P., Naumann M.S. (2016). Impacts of a high-discharge submarine sewage outfall on water quality in the coastal zone of Salvador (Bahia, Brazil). Mar. Pollut. Bull..

[68] Wang Y., Zhang M., Wang X. (2000). Population Growth Responses of Tetrahymena shanghaiensis in Exposure to Rare Earth Elements. Biol. Trace Element Res..

[69] Morroni L., Pinsino A., Pellegrini D., Regoli F., Matranga V. (2016). Development of a new integrative toxicity index based on an improvement of the sea urchin embryo toxicity test. Ecotoxicol. Environ. Saf..

[70] Saitoh M., Kuroda R., Muranaka Y., Uto N., Murai J., Kuroda H. (2010). Asymmetric inhibition of spicule formation in sea urchin embryos with low concentrations of gadolinium ion. Dev. Growth Differ..

[71] Martino C., Bonaventura R., Byrne M., Roccheri M., Matranga V. (2017). Effects of exposure to gadolinium on the development of geographically and phylogenetically distant sea urchins species. Mar. Environ. Res..

[72] Gagné F. (2017). Toxicity and Disruption of Quorum Sensing in Aliivibrio Fisheri by Environmental Chemicals: Impacts of Selected Contaminants and Microplastics. J. Xenobiotics.

[73] Steinberg S.M., Poziomek E.J., Engelmann W.H., Rogers K.R. (1995). A review of environmental applications of bioluminescence measurements. Chemosphere.

[74] Fuma S., Takeda H., Miyamoto K., Yanagisawa K., Inoue Y., Ishii N., Sugai K., Ishii C., Kawabata Z. (2001). Ecological Evaluation of Gadolinium Toxicity Compared with Other Heavy Metals Using an Aquatic Microcosm. Bull. Environ. Contam. Toxicol..

[75] Chieh C. Water Treatment—Chemistry LibreTexts. https://chem.libretexts.org/Bookshelves/Environmental_Chemistry/Supplemental_Modules_Environmental_Chemistry)/Aquatic_Chemistry/Water_Treatment.

[76] Tyagi V., Chopra A., Durgapal N., Kumar A. (2007). Evaluation of Daphnia magna as an indicator of Toxicity and Treatment efficacy of Municipal Sewage Treatment Plant. J. Appl. Sci. Environ. Manag..

[77] Villegas-Navarro A., Romero González M.C., Rosas López E., Domínguez Aguilar R., Sachetin Marçal W. (1999). Evaluation of Daphnia magna as an indicator of toxicity and treatment efficacy of textile wastewaters. Environ. Int..

[78] Elizalde-González M.P., García-Díaz E., González-Perea M., Mattusch J. (2017). Removal of gadolinium-based contrast agents: Adsorption on activated carbon. Environ. Sci. Pollut. Res..

[79] Verplanck P.L., Taylor H.E., Nordstrom D.K., Barber L.B. (2005). Aqueous Stability of Gadolinium in Surface Waters Receiving Sewage Treatment Plant Effluent, Boulder Creek, Colorado. Environ. Sci. Technol..

[80] Lawrence M.G. (2010). Detection of anthropogenic gadolinium in the Brisbane River plume in Moreton Bay, Queensland, Australia. Mar. Pollut. Bull..

[81] Oturan M.A., Aaron J.-J. (2014). Advanced Oxidation Processes in Water/Wastewater Treatment: Principles and Applications. A Review. Crit. Rev. Environ. Sci. Technol..

[82] Niederste-Hollenberg J., Eckartz K., Peters A., Hillenbrand T., Maier U., Beer M., Reszt A. (2018). Reducing the Emission of X-Ray Contrast Agents to the Environment: Decentralized Collection of Urine Bags and Its Acceptance. GAIA Ecol. Perspect. Sci. Soc..

[83] Hu F., Zhao Y.S. (2012). Inorganic nanoparticle-based T1 and T1/T2 magnetic resonance contrast probes. Nanoscale.

[84] Flacke S., Fischer S., Scott M.J., Fuhrhop R.J., Allen J.S., McLean M., Winter P., Sicard G.A., Gaffney P.J., Wickline S.A. (2001). Novel MRI Contrast Agent for Molecular Imaging of Fibrin Implications for Detecting Vulnerable Plaques. Circulation.

[85] Rieter W.J., Kim J.S., Taylor K.M.L., An H., Lin W., Tarrant T., Lin W. (2007). Hybrid Silica Nanoparticles for Multimodal Imaging. Angew. Chem. Int. Ed..

[86] Rieter W.J., Taylor K.M.L., An H., Lin W., Lin W. (2006). Nanoscale Metal−Organic Frameworks as Potential Multimodal Contrast Enhancing Agents. J. Am. Chem. Soc..

[87] Richard C., Doan B.-T., Beloeil J.-C., Bessodes M., Tóth É., Scherman D. (2008). Noncovalent Functionalization of Carbon Nanotubes with Amphiphilic Gd3+Chelates: Toward Powerful T1and T2MRI Contrast Agents. Nano Lett..

[88] Sitharaman B., Kissell K.R., Hartman K.B., Tran L.A., Baikalov A., Rusakova I., Sun Y., Khant H.A., Ludtke S.J., Chiu W. (2005). Superparamagnetic gadonanotubes are high-performance MRI contrast agents. Chem. Commun..

[89] Bin Na H., Lee J.H., An K., Park Y.I., Park M., Lee I.S., Nam D.-H., Kim S.T., Kim S.-H., Kim S.-W. (2007). Development of aT1 Contrast Agent for Magnetic Resonance Imaging Using MnO Nanoparticles. Angew. Chem..

[90] Dubertret M.N.B., Skourides P., Norris D.J., Noireaux V., Brivanlou A.H., Libchaber A. (2002). In Vivo Imaging of Quantum Dots Encapsulated in Phospholipid Micelles. Science.

[91] Gilad A.A., Walczak P., McMahon M.T., Hyon B.N., Jung H.L., An K., Hyeon T., Van Zijl P.C.M., Bulte J.W.M. (2008). MR tracking of transplanted cells with “positive contrast” using manganese oxide nanoparticles. Magn. Reson. Med..

[92] Lee S.-H., Kim B.H., Bin Na H., Hyeon T. (2014). Paramagnetic inorganic nanoparticles as T1 MRI contrast agents. Wiley Interdiscip. Rev. Nanomed. Nanobiotechnol..

[93] Kim T., Momin E., Choi J., Yuan K., Zaidi H., Kim J., Park M., Lee N., McMahon M.T., Quinones-Hinojosa A. (2011). Mesoporous Silica-Coated Hollow Manganese Oxide Nanoparticles as PositiveT1Contrast Agents for Labeling and MRI Tracking of Adipose-Derived Mesenchymal Stem Cells. J. Am. Chem. Soc..

[94] Choi J.Y., Lee S.H., Bin Na H., An K., Hyeon T., Seo T.S. (2010). In vitro cytotoxicity screening of water-dispersible metal oxide nanoparticles in human cell lines. Bioprocess Biosyst. Eng..

[95] Chen R., Ling D., Zhao L., Wang S., Liu Y., Bai R., Baik S., Zhao Y., Chen C., Hyeon T. (2015). Parallel Comparative Studies on Mouse Toxicity of Oxide Nanoparticle- and Gadolinium-Based T1 MRI Contrast Agents. ACS Nano.

[96] Chambon C., Clement O., Le Blanche A., Schouman-Claeys E., Frija G. (1993). Superparamagnetic iron oxides as positive MR contrast agents: In vitro and in vivo evidence. Magn. Reson. Imaging.

[97] Kim B.H., Lee N., Kim H., An K., Park Y.I., Choi Y., Shin K., Lee Y., Kwon S.G., Bin Na H. (2011). Large-Scale Synthesis of Uniform and Extremely Small-Sized Iron Oxide Nanoparticles for High-ResolutionT1Magnetic Resonance Imaging Contrast Agents. J. Am. Chem. Soc..

[98] Song X., Gong H., Yin S., Cheng L., Wang C., Li Z., Li Y., Wang X., Liu G., Liu Z. (2014). Cancer Theranostics: Ultra-Small Iron Oxide Doped Polypyrrole Nanoparticles for In Vivo Multimodal Imaging Guided Photothermal Therapy. Adv. Funct. Mater..

[99] Ibrahim M.A., Dublin A.B. (2020). Magnetic Resonance Imaging (MRI) Gadolinium.

[100] Wang L., Liang T. (2015). Geochemical fractions of rare earth elements in soil around a mine tailing in Baotou, China. Sci. Rep..

[101] Li X., Chen Z., Chen Z., Zhang Y. (2013). A human health risk assessment of rare earth elements in soil and vegetables from a mining area in Fujian Province, Southeast China. Chemosphere.

[102] Adeel M., Lee J.Y., Zain M., Rizwan M., Nawab A., Ahmad M., Shafiq M., Yi H., Jilani G., Javed R. (2019). Cryptic footprints of rare earth elements on natural resources and living organisms. Environ. Int..

[103] Braun M., Zavanyi G., Laczovics A., Berényi E., Szabo S. (2018). Can aquatic macrophytes be biofilters for gadolinium based contrasting agents?. Water Res..

[104] Lingott J., Lindner U., Telgmann L., Esteban-Fernández D., Jakubowski N., Panne U. (2016). Gadolinium-uptake by aquatic and terrestrial organisms-distribution determined by laser ablation inductively coupled plasma mass spectrometry. Environ. Sci. Process. Impacts.

[105] Gwenzi W., Mangori L., Danha C., Chaukura N., Dunjana N., Sanganyado E. (2018). Sources, behaviour, and environmental and human health risks of high-technology rare earth elements as emerging contaminants. Sci. Total. Environ..

[106] Kramsch D.M., Aspen A.J., Apstein C.S. (1980). Suppression of experimental atherosclerosis by the Ca++-antagonist lanthanum. Possible role of calcium in atherogenesis. J. Clin. Investig..

[107] Aime S., Caravan P. (2009). Biodistribution of gadolinium-based contrast agents, including gadolinium deposition. J. Magn. Reson. Imaging.

[108] Leung K. (2012). Clinical use of gadobutrol for contrast-enhanced magnetic resonance imaging of neurological diseases. Rep. Med. Imaging.

[109] Davenport M.S. (2018). Choosing the Safest Gadolinium-based Contrast Medium for MR Imaging: Not So Simple after All. Radiology.

[110] McLachlan S.J., Eaton S., De Simone D.N. (1992). Pharmacokinetic behavior of gadoteridol injection. Investig. Radiol..

[111] Frenzel T., Lengsfeld P., Schirmer H., Hütter J., Weinmann H.-J. (2008). Stability of Gadolinium-Based Magnetic Resonance Imaging Contrast Agents in Human Serum at 37 °C. Investig. Radiol..

[112] Hao D., Ai T., Goerner F., Hu X., Runge V.M., Tweedle M. (2012). MRI contrast agents: Basic chemistry and safety. J. Magn. Reson. Imaging.

[113] McDonald R.J., McDonald J.S., Kallmes D.F., Jentoft M.E., Murray D.L., Thielen K.R., Williamson E.E., Eckel L.J. (2015). Intracranial Gadolinium Deposition after Contrast-enhanced MR Imaging. Radiology.

[114] Errante Y., Cirimele V., Mallio C.A., Di Lazzaro V., Zobel B.B., Quattrocchi C.C. (2014). Progressive Increase of T1 Signal Intensity of the Dentate Nucleus on Unenhanced Magnetic Resonance Images Is Associated With Cumulative Doses of Intravenously Administered Gadodiamide in Patients With Normal Renal Function, Suggesting Dechelation. Investig. Radiol..

[115] Gibby W.A., Gibby K.A., Gibby W.A. (2004). Comparison of Gd DTPA-BMA (Omniscan) versus Gd HP-DO3A (ProHance) Retention in Human Bone Tissue by Inductively Coupled Plasma Atomic Emission Spectroscopy. Investig. Radiol..

[116] Darrah T.H., Prutsman-Pfeiffer J.J., Poreda R.J., Ellen Campbell M., Hauschka P.V., Hannigan R.E. (2009). Incorporation of Excess Gadolinium into Human Bone from Medical Contrast Agents. Metallomics.

[117] Cowper S.E., Robin H.S., Steinberg S.M., Su L.D., Gupta S., LeBoit P.E. (2000). Scleromyxoedema-like Cutaneous Diseases in Renal-Dialysis Patients. Lancet.

[118] Wagner B., Drel V., Gorin Y. (2016). Pathophysiology of Gadolinium-Associated Systemic Fibrosis. Am. J. Physiol. Physiol..

[119] Boyd A.S., Zic J.A., Abraham J.L. (2007). Gadolinium Deposition in Nephrogenic Fibrosing Dermopathy. J. Am. Acad. Dermatol..

[120] High W.A., Ayers R.A., Chandler J., Zito G., Cowper S.E. (2007). Gadolinium Is Detectable within the Tissue of Patients with Nephrogenic Systemic Fibrosis. J. Am. Acad. Dermatol..

[121] Leisman S. (2020). Radiocontrast Toxicity. Adv. Chronic Kidney Dis..

[122] Angeli J.K., Ramos D.B., Casali E.A., Souza D.O.G., Sarkis J.J.F., Stefanon I., Vassallo D.V., Fürstenau C.R. (2011). Gadolinium Increases the Vascular Reactivity of Rat Aortic Rings. Braz. J. Med. Biol. Res..

[123] Escalada A., Navarro P., Ros E., Aleu J., Solsona C., Martín-Satué M. (2004). Gadolinium Inhibition of Ecto-Nucleoside Triphosphate Diphosphohydrolase Activity in Torpedo Electric Organ. Neurochem. Res..

[124] Corot C., Idee J.M., Hentsch A.M., Santus R., Mallet C., Goulas V., Bonnemain B., Meyer D. (1998). Structure-Activity Relationship of Macrocyclic and Linear Gadolinium Chelates: Investigation of Transmetallation Effect on the Zinc-Dependent Metallopeptidase Angiotensin-Converting Enzyme. J. Magn. Reson. Imaging.

[125] Gulani V., Calamante F., Shellock F.G., Kanal E., Reeder S.B. (2017). Gadolinium Deposition in the Brain: Summary of Evidence and Recommendations. Lancet Neurol..

[126] Vergauwen E., Vanbinst A.-M., Brussaard C., Janssens P., De Clerck D., Van Lint M., Houtman A.C., Michel O., Keymolen K., Lefevere B. (2018). Central Nervous System Gadolinium Accumulation in Patients Undergoing Periodical Contrast MRI Screening for Hereditary Tumor Syndromes. Hered. Cancer Clin. Pract..

[127] Lonser R.R., Glenn G.M., Walther M., Chew E.Y., Libutti S.K., Linehan W.M., Oldfield E.H. (2003). Von Hippel-Lindau Disease. Lancet.

[128] Krueger D.A., Northrup H., International Tuberous Sclerosis Complex Consensus Group (2013). Tuberous Sclerosis Complex Surveillance and Management: Recommendations of the 2012 International Tuberous Sclerosis Complex Consensus Conference. Pediatr. Neurol..

[129] Kanda T., Ishii K., Kawaguchi H., Kitajima K., Takenaka D. (2014). High Signal Intensity in the Dentate Nucleus and Globus Pallidus on Unenhanced T1-Weighted MR Images: Relationship with Increasing Cumulative Dose of a Gadoliniumbased Contrast Material. Radiology.

[130] Kanda T., Nakai Y., Hagiwara A., Oba H., Toyoda K., Furui S. (2017). Distribution and Chemical Forms of Gadolinium in the Brain: A Review. Br. J. Radiol..

[131] Radbruch A., Haase R., Kieslich P.J., Weberling L.D., Kickingereder P., Wick W., Schlemmer H.P., Bendszus M. (2017). No Signal Intensity Increase in the Dentate Nucleus on Unenhanced T1-Weighted Mr Images after More than 20 Serial Injections of Macrocyclic Gadolinium-Based Contrast Agents. Radiology.

[132] Jost G., Lenhard D.C., Sieber M.A., Lohrke J., Frenzel T., Pietsch H. (2016). Signal Increase on Unenhanced T1-Weighted Images in the Rat Brain after Repeated, Extended Doses of Gadolinium-Based Contrast Agents Comparison of Linear and Macrocyclic Agents. Investig. Radiol..

[133] Robert P., Lehericy S., Grand S., Violas X., Fretellier N., Ideé J.M., Ballet S., Corot C. (2015). T1-Weighted Hypersignal in the Deep Cerebellar Nuclei after Repeated Administrations of Gadolinium-Based Contrast Agents in Healthy Rats: Difference between Linear and Macrocyclic Agents. Investig. Radiol..

[134] Welk B., McArthur E., Morrow S.A., MacDonald P., Hayward J., Leung A., Lum A. (2016). Association between Gadolinium Contrast Exposure and the Risk of Parkinsonism. JAMA J. Am. Med. Assoc..

[135] Williams S., Grimm H. Gadolinium Toxicity: A Survey of the Chronic Effects of Retained Gadolinium from Contrast MRIs. www.GadoliniumToxicity.com.

[136] Łȩczkowska A., Vilar R. (2013). Interaction of Metal Complexes with Nucleic Acids. Annu. Rep. Prog. Chem. Sect. A.

[137] Kohoutkova V., Babula P., Opatrilova R., Vrana O., Kizek R., Kohoutkova V., Babula P. (2014). Analysis of DNA Modified by Cerium (III), Lanthanum (III) and Gadolinium (III) Ions by Using of Raman Spectroscopy. J. Biochem. Technol..

[138] Kim S.H., Shin W.C., Warrant R.W. (1985). Heavy Metal Ion-Nucleic Acid Interaction. Methods Enzymol..

[139] Jack A., Landner J.E., Rhodes D., Brown R.S., Klug A. (1977). A Crystallographic Study of Metal-Binding to Yeast Phenylalanine Transfer RNA. J. Mol. Biol..

[140] Stout C.D., Mizuno H., Rao S.T., Swaminathan P., Rubin J., Brennan T., Sundaralingam M. (1978). Crystal and Molecular Structure of Yeast Phenylalanyl Transfer RNA. Structure Determination, Difference Fourier Refinement, Molecular Conformation, Metal and Solvent Binding. Acta Crystallogr. Sect. B Struct. Crystallogr. Cryst. Chem..

[141] Angyal S.J. (1972). Complexes of Carbohydrates with Metal Cations: I. Determination of the Extent of Complexing by N.M.R. Spectroscopy. Aust. J. Chem..

[142] Beattie J.K., Terry Kelso M. (1981). Equilibrium and Dynamics of the Binding of Calcium Ion to Sorbitol (D-Glucitol). Aust. J. Chem..

[143] Dill K., Daman M.E., Batstone-Cunningham R.L., Lacombe J.M., Pavia A.A. (1983). 13C-n.m.r.-Spectral Study of the Mode of Binding of Gd3+ to Various Glycopeptides. Carbohydr. Res..

[144] Varki A. (2008). Sialic Acids in Human Health and Disease. Trends Mol. Med..

[145] Zhang Z., Wuhrer M., Holst S. (2018). Serum Sialylation Changes in Cancer. Glycoconj. J..

[146] Dall’Olio F., Chiricolo M. (2001). Sialyltransferases in Cancer. Glycoconj. J..

[147] Schauer R. (2000). Achievements and Challenges of Sialic Acid Research. Glycoconj. J..

[148] Regueiro-Figueroa M., Djanashvili K., Esteban-Gómez D., Chauvin T., Tóth É., De Blas A., Rodríguez-Blas T., Platas-Iglesias C. (2010). Molecular Recognition of Sialic Acid by Lanthanide(III) Complexes through Cooperative Two-Site Binding. Inorg. Chem..

[149] Lacampagne A., Gannier F., Argibay J., Garnier D., Le Guennec J.Y. (1994). The Stretch-Activated Ion Channel Blocker Gadolinium Also Blocks L-Type Calcium Channels in Isolated Ventricular Myocytes of the Guinea-Pig. Biochim. Biophys. Acta BBA Biomembr..

[150] Suzuki S., Toledo-Pereyra L.H., Rodriguez F., Lopez F. (1994). Role of Kupffer Cells in Neutrophil Activation and Infiltration Following Total Hepatic Ischemia and Reperfusion. Circ. Shock.

[151] Callery M.P., Kamei T., Flye M.W. (1990). Kupffer Cell Blockade Increases Mortality During Intra-Abdominal Sepsis Despite Improving Systemic Immunity. Arch. Surg..

[152] Palasz A., Czekaj P. (2000). Toxicological and Cytophysiological Aspects of Lanthanides Action. Acta Biochim. Pol..

[153] Decker K. (1990). Biologically Active Products of Stimulated Liver Macrophages (Kupffer Cells). Eur. J. Biochem..

[154] Rai R.M., Yang S.Q., Mcclain C., Karp C.L., Klein A.S., Diehl A.M. (1996). Kupffer Cell Depletion by Gadolinium Chloride Enhances Liver Regeneration after Partial Hepatectomy in Rats. Am. J. Physiol. Gastrointest. Liver Physiol..

[155] Rüttinger D., Vollmar B., Wanner G.A., Messmer K. (1996). In Vivo Assessment of Hepatic Alterations Following Gadolinium Chloride-Induced Kupffer Cell Blockade. J. Hepatol..

[156] Jarrar D., Wang P., Chaudry I.H., Holzheimer R., Mannick J. (2001). Hepatocellular Dysfunction–Basic Considerations. Surgical Treatment: Evidence-Based and Problem-Oriented.

[157] Ryder K.W., Kaplan J.E., Saba T.M. (1975). Serum Calcium and Hepatic Kupffer Cell Phagocytosis. Exp. Biol. Med..

[158] Abdel-Razzak Z., Loyer P., Fautrel A., Gautier J.C., Corcos L., Turlin B., Beaune P., Guillouzo A. (1993). Cytokines Down-Regulate Expression of Major Cytochrome P-450 Enzymes in Adult Human Hepatocytes in Primary Culture. Mol. Pharmacol..

[159] Badger D.A., Kuester R.K., Sauer J.M., Sipes I.G. (1997). Gadolinium Chloride Reduces Cytochrome P450: Relevance to Chemical-Induced Hepatotoxicity. Toxicology.

[160] Kanda T., Oba H., Toyoda K., Kitajima K., Furui S. (2016). Brain Gadolinium Deposition after Administration of Gadolinium-Based Contrast Agents. Jpn. J. Radiol..

[161] Barbieri S., Schroeder C., Froehlich J.M., Pasch A., Thoeny H.C. (2016). High Signal Intensity in Dentate Nucleus and Globus Pallidus on Unenhanced T1-Weighted MR Images in Three Patients with Impaired Renal Function and Vascular Calcification. Contrast Media Mol. Imaging.

[162] Huettner J.E., Stack E., Wilding T.J. (1998). Antagonism of Neuronal Kainate Receptors by Lanthanum and Gadolinium. Neuropharmacology.

[163] Chittajallu R., Vignes M., Dev K.K., Barnes J.M., Collingridge G.L., Henley J.M. (1996). Regulation of Glutamate Release by Presynaptic Kainate Receptors in the Hippocampus. Nature.

[164] Castillo P.E., Malenka R.C., Nicoll R.A. (1997). Kainate Receptors Mediate a Slow Postsynaptic Current in Hippocampal CA3 Neurons. Nature.

[165] Monaghan D.T., Bridges R.J., Cotman C.W. (1989). The Excitatory Amino Acid Receptors: Their Classes, Pharmacology, and Distinct Properties in the Function of the Central Nervous System. Annu. Rev. Pharmacol. Toxicol..

[166] Reichling D.B., MacDermott A.B. (1991). Lanthanum Actions on Excitatory Amino Acid-gated Currents and Voltage-gated Calcium Currents in Rat Dorsal Horn Neurons. J. Physiol..

[167] Sutresno A., Haryanto F., Viridi S., Arif I. (2020). Influence Blocking by Gadolinium in Calcium Diffusion on Synapse Model: A Monte Carlo Simulation Study. J. Biomed. Phys. Eng..

[168] Sachs F. (2010). Stretch-Activated Ion Channels: What Are They?. Physiology.

[169] Millet B., Pickard B.G. (1988). Gadolinium Ion Is an Inhibitor Suitable for Testing the Putative Role of Stretch-Activated Ion Channels in Geotropism and Thigmotropism. Biophys. J..

[170] Yang X.C., Sachs F. (1989). Block of Stretch-Activated Ion Channels in Xenopus Oocytes by Gadolinium and Calcium Ions. Science.

[171] Neher E., Steinbach J.H. (1978). Local Anaesthetics Transiently Block Currents through Single Acetylcholine-receptor Channels. J. Physiol..

[172] Dani J.A. (1986). Ion-Channel Entrances Influence Permeation. Net Charge, Size, Shape, and Binding Considerations. Biophys. J..

[173] Gustin M.C., Zhou X.L., Martinac B., Kung C. (1988). A Mechanosensitive Ion Channel in the Yeast Plasma Membrane. Science.

[174] Alba A., Kano M., Chen C., Stanton M.E., Fox G.D., Herrup K., Zwingman T.A., Tonegawa S. (1994). Deficient Cerebellar Long-Term Depression and Impaired Motor Learning in MGluR1 Mutant Mice. Cell.

[175] Hermans E., Challiss R.A.J. (2001). Structural, Signalling and Regulatory Properties of the Group I Metabotropic Glutamate Receptors: Prototypic Family C G-Protein-Coupled Receptors. Biochem. J..

[176] Tateyama M., Kubo Y. (2006). Dual Signaling Is Differentially Activated by Different Active States of the Metabotropic Glutamate Receptor 1α. Proc. Natl. Acad. Sci. USA.

[177] Kovacs G., Danko T., Bergeron M.J., Balazs B., Suzuki Y., Zsembery A., Hediger M.A. (2011). Heavy Metal Cations Permeate the TRPV6 Epithelial Cation Channel. Cell Calcium.

[178] Südhof T.C. (2004). The Synaptic Vesicle Cycle. Annu. Rev. Neurosci..

[179] Van Meer G., Voelker D.R., Feigenson G.W. (2008). Membrane Lipids: Where They Are and How They Behave. Nat. Rev. Mol. Cell Biol..

[180] Crane J.M., Tamm L.K. (2004). Role of Cholesterol in the Formation and Nature of Lipid Rafts in Planar and Spherical Model Membranes. Biophys. J..

[181] Singer S.J., Nicolson G.L. (1972). The Fluid Mosaic Model of the Structure of Cell Membranes. Science.

[182] Edidin M. (2003). Lipids on the Frontier: A Century of Cell-Membrane Bilayers. Nat. Rev. Mol. Cell Biol..

[183] Zachowski A. (1993). Phospholipids in Animal Eukaryotic Membranes: Transverse Asymmetry and Movement. Biochem. J..

[184] Kay J.G., Fairn G.D. (2019). Distribution, Dynamics and Functional Roles of Phosphatidylserine within the Cell. Cell Commun. Signal..

[185] Boettcher J.M., Davis-Harrison R.L., Clay M.C., Nieuwkoop A.J., Ohkubo Y.Z., Tajkhorshid E., Morrissey J.H., Rienstra C.M. (2011). Atomic View of Calcium-Induced Clustering of Phosphatidylserine in Mixed Lipid Bilayers. Biochemistry.

[186] Cullis P.R., De Kruijff B. (1979). Lipid Polymorphism and the Functional Roles of Lipids in Biological Membranes. Biochim. Biophys. Acta BBA Rev. Biomembr..

[187] Lewis R.N.A.H., McElhaney R.N. (2013). Membrane Lipid Phase Transitions and Phase Organization Studied by Fourier Transform Infrared Spectroscopy. Biochim. Biophys. Acta BBA Biomembr..

[188] Sule K., Umbsaar J., Prenner E.J. (2020). Mechanisms of Co, Ni, and Mn Toxicity: From Exposure and Homeostasis to Their Interactions with and Impact on Lipids and Biomembranes. Biochim. Biophys. Acta BBA Biomembr..

[189] Evans C.H. (1990). Membrane Interactions of Lanthanide Ions. Biochemistry of the Lanthanides.

[190] Payliss B.J., Hassanin M., Prenner E.J. (2015). The Structural and Functional Effects of Hg(II) and Cd(II) on Lipid Model Systems and Human Erythrocytes: A Review. Chem. Phys. Lipids.

[191] Li X.-M., Zhang Y.-F., Ni J.-Z., Chen J.-W., Hwang F. (1994). Effect of Lanthanide Ions on the Phase Behavior of Dipalmitoylphosphatidylcholine Multilamellar Liposomes. J. Inorg. Biochem..

[192] Sabín J., Prieto G., Blanco E., Ruso J.M., Angelini R., Bordi F., Sarmiento F. (2007). Interaction of Gadolinium with Phospholipids Bilayer Membranes: Photon Correlation Spectroscopy and DSC Study. J. Therm. Anal. Calorim..

[193] Tanaka T., Tamba Y., Masum S.M., Yamashita Y., Yamazaki M. (2002). La3+ and Gd3+ Induce Shape Change of Giant Unilamellar Vesicles of Phosphatidylcholine. Biochim. Biophys. Acta BBA Biomembr..

[194] Averbakh A., Pavlov D., Lobyshev V.I. (2000). Effect of Gadolinium(III) Ions on the Phase Behaviour of Dimyristoylphosphatidyl Serine Multilamellar Liposomes. J. Therm. Anal. Calorim..

[195] Gianulis E.C., Pakhomov A.G. (2015). Gadolinium Modifies the Cell Membrane to Inhibit Permeabilization by Nanosecond Electric Pulses. Arch. Biochem. Biophys..

[196] Cheng Y., Liu M., Li R., Wang C., Bai C., Wang K. (1999). Gadolinium Induces Domain and Pore Formation of Human Erythrocyte Membrane: An Atomic Force Microscopic Study. Biochim. Biophys. Acta BBA Biomembr..

[197] Lopatina L.P., Waseem T.W., Fedorovich S.V., Konev S.V. (2005). Lanthanides Induce Neurotransmitter Release from the Vesicular Pool in Rat Brain Synaptosomes. Biofizika.

[198] Cosgrove T. (2010). Colloid Science: Principles, Methods, and Applications.

[199] McLaughlin S., Mulrine N., Gresalfi T., Vaio G., McLaughlin A. (1981). Adsorption of Divalent Cations to Bilayer Membranes Containing Phosphatidylserine. J. Gen. Physiol..

[200] Ermakov Y.A., Averbakh A.Z., Arbuzova A.B., Sukharev S.I. (1997). Lipid and Cell Membranes in the Presence of Gadolinium and Other Ions with High Affinity to Lipids. 2. A Dipole Component of the Boundary Potential on Membranes with Different Surface Charges. Membr. Cell Biol..

[201] Ermakov Y.A., Averbakh A.Z., Sukharev S.I. (1997). Lipid and Cell Membranes in the Presence of Gadolinium and Other Ions with High Affinity to Lipids. 1. Dipole and Diffuse Components of the Boundary Potential. Membr. Cell Biol..

[202] Hahne H.C.H., Kroontje W. (1973). Significance of PH and Chloride Concentration on Behavior of Heavy Metal Pollutants: Mercury(II), Cadmium(II), Zinc(II), and Lead(II). J. Environ. Qual..

[203] Kerek E., Hassanin M., Prenner E.J. (2018). Inorganic Mercury and Cadmium Induce Rigidity in Eukaryotic Lipid Extracts While Mercury Also Ruptures Red Blood Cells. Biochim. Biophys. Acta BBA Biomembr..

[204] Gustafsson J.P. (2016). Visual MINTEQ Chemical Equilibrium Software.

[205] Kerek E.M., Prenner E.J. (2016). Inorganic Cadmium Affects the Fluidity and Size of Phospholipid Based Liposomes. Biochim. Biophys. Acta BBA Biomembr..

[206] Kerek E., Hassanin M., Zhang W., Prenner E.J. (2017). Preferential Binding of Inorganic Mercury to Specific Lipid Classes and Its Competition with Cadmium. Biochim. Biophys. Acta BBA Biomembr..

[207] Parasassi T., De Stasio G., Ravagnan G., Rusch R.M., Gratton E. (1991). Quantitation of Lipid Phases in Phospholipid Vesicles by the Generalized Polarization of Laurdan Fluorescence. Biophys. J..

[208] ATSDR Substance Priority List. https://www.atsdr.cdc.gov/spl/index.html#2019spl.

[209] Baba T., Campbell J.L., Le Blanc J.C.Y., Baker P.R.S. (2016). In-Depth Sphingomyelin Characterization Using Electron Impact Excitation of Ions from Organics and Mass Spectrometry. J. Lipid Res..

[210] Prenner E., Honsek G., Hönig D., Möbius D., Lohner K. (2007). Imaging of the Domain Organization in Sphingomyelin and Phosphatidylcholine Monolayers. Chem. Phys. Lipids.

[211] Lingwood D., Simons K. (2010). Lipid Rafts as a Membrane-Organizing Principle. Science.

[212] Le M.T., Hassanin M., Mahadeo M., Gailer J., Prenner E.J. (2013). Hg- and Cd-Induced Modulation of Lipid Packing and Monolayer Fluidity in Biomimetic Erythrocyte Model Systems. Chem. Phys. Lipids.

[213] Amoruso M.A., Witz G., Goldstein B.D. (1987). Alteration of Erythrocyte Membrane Fluidity by Heavy Metal Cations. Toxicol. Ind. Health.

[214] Suwalsky M., Villena F., Norris B., Cuevas F., Sotomayor C.P. (2004). Cadmium-Induced Changes in the Membrane of Human Erythrocytes and Molecular Models. J. Inorg. Biochem..

[215] Lee W.K., Probst S., Santoyo-Sánchez M.P., Al-Hamdani W., Diebels I., von Sivers J.K., Kerek E., Prenner E.J., Thévenod F. (2017). Initial Autophagic Protection Switches to Disruption of Autophagic Flux by Lysosomal Instability during Cadmium Stress Accrual in Renal NRK-52E Cells. Arch. Toxicol..

[216] Jeffery E.H., Abreo K., Burgess E., Cannata J., Greger J.L. (1996). Systemic Aluminum Toxicity: Effects on Bone, Hematopoietic Tissue, and Kidney. J. Toxicol. Environ. Health.

[217] Zatta P., Kiss T., Suwalsky M., Berthon G. (2002). Aluminium(III) as a Promoter of Cellular Oxidation. Coord. Chem. Rev..

[218] Verstraeten S.V., Oteiza P.I. (1995). Sc3+, Ga3+, In3+, Y3+, and Be2+ Promote Changes in Membrane Physical Properties and Facilitate Fe2+-Initiated Lipid Peroxidation. Arch. Biochem. Biophys..

[219] Verstraeten S.V., Aimo L., Oteiza P.I. (2008). Aluminium and Lead: Molecular Mechanisms of Brain Toxicity. Arch. Toxicol..

[220] Adonaylo V.N., Oteiza P.I. (1999). Pb2+ Promotes Lipid Oxidation and Alterations in Membrane Physical Properties. Toxicology.

[221] Althumairy D., Murakami H.A., Zhang D., Barisas B.G., Roess D.A., Crans D.C. (2020). Effects of Vanadium(IV) Compounds on Plasma Membrane Lipids Lead to G Protein-Coupled Receptor Signal Transduction. J. Inorg. Biochem..

[222] Levina A., Crans D.C., Lay P.A. (2017). Speciation of Metal Drugs, Supplements and Toxins in Media and Bodily Fluids Controls in Vitro Activities. Coord. Chem. Rev..

[223] Crans D.C., Woll K.A., Prusinskas K., Johnson M.D., Norkus E. (2013). Metal Speciation in Health and Medicine Represented by Iron and Vanadium. Inorg. Chem..

